# Innovative HPMC/PVP K90 Dissolving Microneedles Incorporating Tacrolimus-Loaded Cubosomes: A Novel Strategy for Managing Allergic Conjunctivitis

**DOI:** 10.3390/pharmaceutics18040459

**Published:** 2026-04-09

**Authors:** Sammar Fathy Elhabal, Mai S. Shoela, Fatma E. Hassan, Suzan Awad AbdelGhany Morsy, Shady Allam, Reem Abd Elhameed Aldeeb, Amal Anwar Taha, Rania Mostafa Abd El Galil, Amr M. Emam, Nahla A. Elzohairy, Hanaa Wanas, Ahmed Mohsen Elsaid Hamdan

**Affiliations:** 1 Department of Pharmaceutics and Industrial Pharmacy, Faculty of Pharmacy, Modern University for Technology and Information (MTI), Mokattam, Cairo 11571, Egypt; 2 Department of Clinical Pharmacology, Faculty of Medicine, Alexandria University, Alexandria 21526, Egypt; 3 Department of Physiology, General Medicine Practice Program, Batterjee Medical College, Jeddah 21442, Saudi Arabia; 4 Medical Physiology Department, Faculty of Medicine, Cairo University, Kasr Alainy, Giza 11562, Egypt; 5 Pathological Sciences Department, MBBS Program, Fakeeh College for Medical Sciences, Jeddah 21461, Saudi Arabia; 6 Department of Pharmacology and Toxicology, Faculty of Pharmacy, Menoufia University, Menoufia 32811, Egypt; 7 Department of Pharmacology and Toxicology, Faculty of Pharmacy, Menoufia National University, Cairo-Alexandria Agricultural Road, Menoufia 32511, Egypt; 8 Department of Pharmaceutics, College of Pharmaceutical Sciences and Drug Manufacturing, Misr University for Science and Technology, 6th of October City 12566, Egypt; 9 Department of Pharmacology and Toxicology, College of Pharmaceutical Sciences and Drug Manufacturing, Misr University for Science and Technology, 6th of October City 12566, Egypt; 10 Department of Microbiology and Immunology, Faculty of Pharmacy, Modern University for Technology and Information (MTI), Mokattam, Cairo 11571, Egypt; 11 Air Force Specialized Hospital, Cairo 19448, Egypt; 12 Medical Pharmacology Department, Faculty of Medicine, Cairo University, Cairo 11956, Egypt; 13 Pharmacology and Toxicology Department, Faculty of Pharmacy, Taibah University, Madinah 41477, Saudi Arabia; 14 Department of Pharmacology and Toxicology, Faculty of Pharmacy, University of Tabuk, Tabuk 71491, Saudi Arabia; 15 Prince Fahad bin Sultan Chair for Biomedical Research (PFSCBR), Tabuk 74191, Saudi Arabia

**Keywords:** tacrolimus, cubosomes, dissolving microneedles, ocular drug delivery, allergic conjunctivitis, controlled release, anti-inflammatory therapy

## Abstract

**Background/Objectives**: Allergic conjunctivitis (AC) is the most common inflammatory disease affecting the ocular conjunctiva. Tacrolimus (TCR), a potent calcineurin inhibitor, is limited by poor aqueous solubility and low ocular bioavailability. This study aimed to develop TCR-loaded cubosomes (TCR-Cubs) incorporated into HPMC/PVP K90 dissolving microneedles (MNs) to enhance their therapeutic efficacy. **Methods**: TCR-Cubs were prepared using a modified top-down fragmentation method with glyceryl monooleate and poloxamer 407, optimized via Box–Behnken design, and incorporated into dissolving MNs. The system was evaluated in vitro, ex vivo, and in vivo using a rabbit model of allergic conjunctivitis. **Results**: The optimized formulation exhibited the smallest particle size (210 ± 0.91 nm), polydispersity index (0.29 ± 0.03), zeta potential (−21 ± 0.87 mV), and the highest entrapment efficiency (% 93.3 ± 0.45). The optimized formulation was incorporated into MNs via micro molding. Scanning electron microscopy (SEM) confirmed well-defined, sharp microneedles, with low height reduction (<10%) by mechanical testing and high penetration efficiency (>85–90%). In vitro release studies revealed sustained drug release of (~75–80%) over 24 h, compared to (~40%) from the TCR suspension, following diffusion-controlled kinetics. Ex vivo permeation studies showed a (~2–3-fold) enhancement in corneal drug flux. In vivo pharmacodynamic evaluation using an ovalbumin-induced allergic conjunctivitis model demonstrated significant reductions in inflammatory mediators, including inflammatory markers (TNF-α, IL-1β, IL-6, NLRP3), which were reduced by (~50–75%), with modulation of CPA3, BCL2, and TGF-β1 by qRT-PCR. Histopathology and TLR4 analysis confirmed reduced inflammation without irritation. **Conclusions**: This dual-delivery system offers a promising, non-invasive platform for enhanced ocular delivery of tacrolimus with superior anti-inflammatory efficacy in allergic conjunctivitis.

## 1. Introduction

Allergic conjunctivitis (AC) exemplifies an IgE-mediated hypersensitivity reaction. In response to an environmental allergen, the body triggers mast cell degranulation, leading to the release of histamine and subsequent conjunctival hyperemia, pruritus, lacrimation, and, ultimately, chronic inflammatory changes [1,2]. Over six million Americans of all ages and walks of life have conjunctivitis, the leading cause of red eye. Frontline therapy for allergic conjunctivitis consists of both topical antihistamine and mast cell-stabilizing eye drops [3,4]. Because combined antihistamine formulations can relieve pruritus and redness while inhibiting mast cell degranulation, they are the preferred choice for ocular therapy. Pure antihistaminic drops (e.g., amaprine, emedastine) are relatively ineffective for providing anti-pruritic and anti-redness relief, which is why they are used in conjunction with prophylactic mast cell stabilizers (e.g., sodium cromolyn, ketotifen fumarate, or nedocromil), which are used for longer-term treatment and act much more slowly; and are used more as a treatment to prevent allergic conjunctivitis [1,5]. In more severe or refractory cases (such as vernal and atopic keratoconjunctivitis), a short course of topical corticosteroids, such as prednisolone acetate, dexamethasone, loteprednol, or fluocinolone, may be required. However, because prolonged use of these medications may result in serious adverse effects, patients must be evaluated regularly to identify early signs of complications (such as ocular hypertension, cataracts, and infections) [6,7]. For patients with chronic, severe, steroid-dependent allergic eye disease, calcineurin inhibitors have been shown to reduce the need for steroids and, therefore, type II and type IV closed (cavity) skin through free immunomodulation [8,9]. First, this kind of immunomodulation is highly effective because it targets the T-cell response. Furthermore, the topical administration of tacrolimus ointment (0.03%) to the eyelid margins or conjunctival sac is intended to reduce T-cell inflammation and therefore reduce the need for corticosteroids and control the symptoms of vernal and atopic keratoconjunctivitis [8,10,11]. Additionally, the long-term use of cyclosporine A emulsion or drop formulations has been shown to improve ocular surface inflammation and the stability of the tear film. An iron-binding glycoprotein, Lactoferrin, exhibits anti-inflammatory, antimicrobial, and immunomodulatory effects, and lower levels of lactoferrin are related to ocular surface disease, making adjunct or alternative therapy possible. While it is too early to reach any conclusions, initial exploratory research suggests that the lactoferrin component could alleviate and reduce inflammation in dry and allergic eye disease, as well as in the tear film. This is not to imply that her contribution is fully developed compared to the options offered by antihistamines and calcineurin inhibitors [12,13].

Tacrolimus (TCR) (FK506) is a 23-membered cyclic macrolide lactone with immunomodulatory function derived from the bacteria Streptomyces tsukubensis in 1984 [14,15]. TCR is a topical calcineurin inhibitor that acts as a steroid-sparing immunomodulator for serious or refractory cases of allergic and immune-mediated ocular surface disorders, particularly vernal and atopic keratoconjunctivitis. It inhibits T cell calcineurin, binds to FKBP-12, thereby blocking NF-AT-dependent transcription of cytokines such as IL-2, and reduces T cell, mast cell, and inflammatory mediator release from the eye [16,17]. TCR is classified as a Biopharmaceutics Classification System (BCS) class II drug, with low solubility and high permeability [18]. However, TCR’s physicochemical characteristics (high molecular weight (822.95 g/mol); higher lipophilicity (partition coefficient log P = 3.96 ± 0.83)) indicate that it is unable to permeate the dermal layers of the skin beyond the stratum corneum [19]. Previous studies showed that dermal application of TCR resulted in most of the drug remaining in the stratum corneum, with limited dermal penetration. There is hence a need for a new delivery system for TCR to facilitate penetration into the epidermal and dermal target layers beyond the stratum corneum [20,21]. Currently under investigation are several delivery mechanisms, including polymeric micelle nanocarriers, Solid Lipid Nanoparticles (SLNs), and other nanocarriers. However, the absence of reference products has prevented addressing issues such as poor solubility and high lipophilicity. When treating conjunctivitis, anti-allergic medications are often administered topically because of factors such as ease of administration, non-invasiveness, rapid onset, cost-efficiency, minimal systemic side effects, and high patient compliance [22,23,24]. Topically administered medications can diffuse into various ocular tissues, with the main pathway being the transcorneal route. The nature of the cornea and the poor physicochemical properties of the active drug substances present a challenge to achieving the desired antifungal bioavailability. In response to such challenges, a few non-conventional drug delivery systems have been developed, including lipid nanoparticles, microemulsions, Spanlastics, micelles, and in situ gels [19,25,26].

Recently, MO-based nanosystems, like cubosomes (Cubs), have been gaining attention in ocular drug delivery [27,28]. These cubosomes attain stability after forming a polymeric outer corona, which drives them towards a polymeric corona structure. Due to their large membrane and surface area and ability to achieve optimal drug loading compared to liposomes, cubosomes present promising biocompatibility and drug stability while providing controlled release, maintaining a high level of bioadhesion, and transcorneal permeability with prolonged corneal retention [29,30]. Cubosomes stabilized by nonionic block copolymers, such as Pluronic F127, form a polymeric corona that enhances colloidal stability and inhibits aggregation, surpassing conventional surfactants [31,32,33]. This field has experienced a significant increase in popularity due to recent developments in polymer cubosomes utilizing polystyrene scaffolds, poly (ionic liquid) block copolymers, and photocleavable variations for adjustable release and enhanced loading of hydrophobic pharmaceuticals. These designs provide bicontinuous channels for prolonged administration, suitable for BCS Class II medicines such as tacrolimus [34,35,36].

Microneedles (MNs), on the other hand, are more rigid structures with well-defined heights and spacing [1,37]. The use of microneedles (MNs) avoids contact with nerve fibers and blood vessels in the dermis, which can help alleviate pain. These concerns, along with adverse pain reactions, compliance, and treatment efficacy, have led to the development of MNs [38,39]. MNs can be made from various materials, most commonly metals, polymers, and silica. MNs can be classified into five different types depending on the mechanisms of delivery: solid, hollow, coated, dissolving, and hydrogel-forming MNs [40,41]. Dissolving MNs are made from biocompatible and non-toxic polymers that are soluble in water (e.g., hyaluronic acid, maltose, polyvinyl pyrrolidone, sucrose, and hydroxypropyl methylcellulose) [42]. These polymers are safe for use in the human body [43,44]. The active pharmaceutical ingredients (APIs) contained within dissolving MNs are either fully dissolved or uniformly dispersed within the MNs. In order to provide effective drug delivery, the MNs are designed to fully dissolve after insertion into the skin because the MNs are made from materials that will alter their structure, and water or other bodily fluids (e.g., interstitial fluid) will facilitate the MNs to dissolve and the APIs to be released transdermally, thereby leaving the MNs’ original shape behind. In particular, dissolving MNs have a great deal of versatility and have been designed to incorporate a wide variety of APIs and macromolecules (e.g., DNA, RNA, and proteins) as well as even deliver the large molecules donepezil hydrochloride and propranolol hydrochloride [45,46,47]. 3D printing and micro-electromechanical systems/micromachining (MEMS) have both been used to fabricate MN patches. Though the MEMS technique has the possibility for mass production and replication, it is very labor-intensive and requires a lot of training to learn the complicated multi-step process, and is, therefore, not as suitable as a 3D printing method [48,49].

To address these limitations, our study introduces cubosomes, which have previously been used as drug delivery systems, and, when combined with microneedles, provide a non-invasive and patient-friendly method for drug delivery. The dual delivery system, incorporating microneedles and cubosomes, is designed to deliver TCR (targeted cancer therapy) to the conjunctival space to inhibit conjunctivitis-causing inflammation and achieve higher drug concentrations in the conjunctiva, thereby increasing therapeutic effect and reducing adverse effects from systemic circulation. The results from the Box–Behnken optimization of the TCR cubosomes provided a quality statistical distribution of the size parameters, poly dispersity, zeta potential, and entrapment to design a microneedle system with pre-determined parameters for penetration, drug load, dissolution, and structural integrity determined by standard tests of Scanning Electron Microscopy (SEM), Differential Scanning Calorimetry (DSC), and Fourier Transform Infrared Spectroscopy (FTIR). The purpose of this study was to evaluate the effectiveness of the new design to deliver TCR in the form of TCR-Cubosomes and TCR-Cub/HPMC/PVP K90- MNs for the first time in vivo to an allergically non-infectious conjunctivitis model (AC) in rabbits. Quantified the gene expression levels of BCL2-like 1/BCL2L1 (BCL2) and carboxypeptidase A3 (CPA3) and transforming growth factor beta 1 (TGF-β1) using qRT-PCR, and the tumor necrosis factor-alpha (TNF-α), interleukin-1 beta (IL-1β), interleukin-6 (IL-6), and leucine-rich repeat and pyrin domain containing protein 3 (NLRP3) using ELISA. The histopathological evaluation and TLR4 immunohistochemistry of the proposed TCR-Cub/HPMC/PVP K90- MNs TLR4 give us the potential for future clinical use.

## 2. Materials and Methods

### 2.1. Chemicals and Reagents

Tacrolimus (TCR), glyceryl monooleate (GMO; type I, ≥99% purity), poloxamer 407 (Pluronic^®^ F127; pharmaceutical grade), hydroxypropyl methylcellulose (HPMC E50), polyvinylpyrrolidone K-30 (PVP K-30; average MW ≈ 40,000 Da), and polyvinyl alcohol (PVA; average MW ≈160,000 Da, 88–89% hydrolyzed) were purchased from Sigma-Aldrich (St. Louis, MO, USA). Ovalbumin (OVA, Grade V) and aluminum hydroxide (AH) were also obtained from Sigma-Aldrich (St. Louis, MO, USA). Spectra/Por^®^ regenerated cellulose dialysis membrane tubing (molecular weight cut-off 12,000–14,000 Da) was obtained from Spectrum Laboratories Inc. (Rancho Dominguez, CA, USA). Chloroform, ethanol, acetonitrile, and methanol (HPLC grade) were supplied by Thermo Fisher Scientific (Waltham, MA, USA).

### 2.2. Animals

Thirty male adult albino rabbits were used, weighing about 2.5 ± 0.5 kg. The rabbits were housed in individual cages at 25 ± 2 °C with an alternating 12 h light/dark cycle. The rabbits had access to standard commercial rabbit food and tap water. Prior to the start of the study, all rabbits’ eyes were examined, and only those without ocular inflammation were included. The research was approved by the Research Ethics Committee (REC) of the Faculty of Pharmacy, Cairo University (approval number: PI 4000). The study adhered to the guidelines of the US National Institute of Health (NIH Publication No. 85–23, revised 2011) and the Guide for Care and Use of Laboratory Animals.

### 2.3. Methods

#### 2.3.1. Tacrolimus High-Performance Liquid Chromatography Analysis

Tacrolimus can be analyzed and quantified on a C18 reversed-phase column (e.g., 250 × 4.6 mm, 5 µm ODS-Hypersil) using an isocratic mobile phase of acetonitrile: water (70:30, *v*/*v*) at an appropriate flow rate (about 1.1 mL/min) and UV detection at 220 nm, where TCR shows good absorbance. A sample volume of 20 μL was injected, and chromatography was performed at a flow rate of 1.2 mL/min. Detection was performed with a diode-array detector (DAD)(Agilent Technologies, Waldbronn, Germany) at 300 nm, with the column temperature maintained at 25 °C. The TCR concentrations were determined from a standard calibration curve. The calibration curve ranged from 20 to 100 ng/mL, and recovery ranged from 97.13% to 102.04%. The linearity ranged from 20 to 100 ng/mL (R^2^ = 0.999; *n* = 3) [50,51,52]. Pluronic F127 was chosen as the stabilizer due to its demonstrated effectiveness for GMO-based cubosomes, providing an ideal hydrophilic lipophilic balance (HLB) of 22, a low critical micelle concentration (CMC) of 0.8% wt, a high PPO-block affinity for lipid bilayers (partition coefficient > 50 × 10^4^), and confirmed biocompatibility. F127 concentrations (0.5–2.5% *w*/*w*) were far below the crucial gelation threshold (>20% *w*/*w*), hence maintaining dilute solution behavior [53]. GMO (1.25–10% *w/w*) functioned as a hydrotrope, further inhibiting F127 gelation while generating the bicontinuous cubic phase.

#### 2.3.2. Experimental Design Tacrolimus-Loaded Cubosomes (TCR-Cubs)

A Box–Behnken design was chosen to statistically optimize the independent variables: A: GMO W/W%, B: GMO: F127, and C: Homogenization time. The specified dependent variables were set to Y1: EE (%), Y2: PS (nm), Y3: PDI, and Y4: ZP (mV), as listed in Table 1. The Design Expert^®^ software (Version 13, Stat-Eae Inc., Minneapolis, MN, USA) was used to create 15 formulations; the respective compositions are given in Table 2. For the remaining equations, we have excluded non-significant terms to improve the interpretation of the correlation among the studied and observed variables. Additionally, we have set boundaries on the desired variables to enable the software, using maximum numerical desirability, to obtain the optimal formula [54,55].

#### 2.3.3. Preparation of Tacrolimus-Loaded Cubosomes (TCR-Cubs)

**Figure 1:**
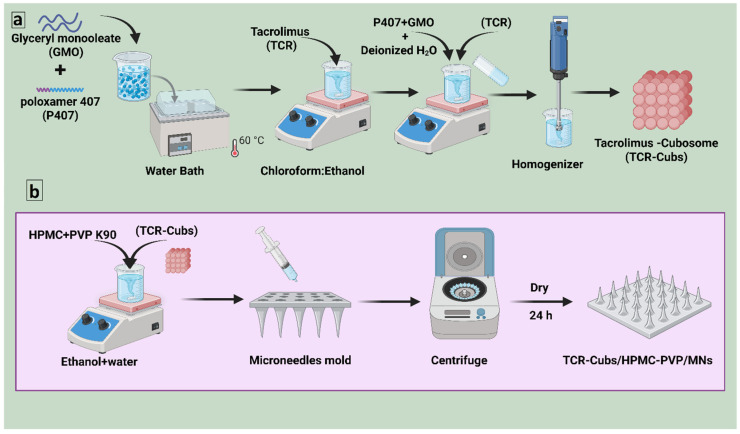
(**a**) Schematic diagram illustrating the methodology for preparing tacrolimus-loaded cubosomes (TCR-Cubs), and (**b**) schematic diagram illustrating the methodology for fabrication of optimized TCR-Cubs-loaded HPMC-PVP K90 microneedle patches (TCR-Cubs/HPMC-PVP K90/MNs).

Tacrolimus-loaded cubosomes were prepared by using a modified top-down fragmentation method with glyceryl monooleate (GMO) and poloxamer 407 (P407) as the lipid and stabilizer, respectively. The precise amounts of GMO and P407 were calculated according to the experimental design in Table 1, and the mixture was melted in a water bath at 60 °C until a clear, homogeneous lipid phase was obtained. Tacrolimus (10 mg) was dissolved in chloroform: ethanol (2:1, *v*/*v*), heated to 60 °C, and then added to the molten GMO/P407 mixture while stirring to achieve uniform dispersion of the drug. The organic solvents were removed by evaporation in a 60 °C water bath until a viscous drug-loaded lipid phase was obtained. Deionized water (2 mL), pre-heated to 40–60 °C, was added dropwise to the molten lipid phase while stirring to create a cubic gel. The gel was then equilibrated at room temperature for 48 h to ensure complete formation of the cubic phase. Following equilibration, the bulk cubic gel was diluted with the remaining required volume of deionized water and dispersed by vortex mixing to obtain a coarse cubosomal dispersion. To break down the bulk cubic phase into cubosomal nanoparticles, the final coarse dispersion was ultrasonicated using a probe at 60% amplitude. This process consisted of 5 s of sonication followed by 5 s of pause, repeated 0, 5, or 10 times, depending on the formulation. Once formed, the tacrolimus-loaded cubosomes were packaged into vials and stored at room temperature until characterization could begin [56,57]. The tacrolimus-loaded cubosomes formulations (F1–F15) were made by varying the GMO concentration at 1.25, 5.0, and 10.0% *w*/*w* of the total dispersion, and varying the P407/GMO ratio at (5, 15, and 25% *w*/*w*), keeping the amount of tacrolimus at 10 mg constant in all the formulations, as shown in Figure 1a.

#### 2.3.4. In Vitro Characterization of Tacrolimus-Loaded Cubosomes (TCR-Cubs)

##### Determination of Particle Size (PS), Polydispersity Index (PDI), and Zeta Potential (ZP)

The physicochemical properties of tacrolimus-loaded Cubs were assessed in terms of, polydispersity index (PDI), and zeta (Z) potential, the cubosomal dispersion of TCR was analyzed at a temperature of 25 °C ± 2 °C, using an aliquot of 0.1 mL, which was then subjected to a 100-fold dilution using double distilled water to a concentration that achieves appropriate scattering intensity to be measured by the Zetasizer Nano ZS (Malvern Instruments; Worcestershire, UK). This methodology is based on the theory of dynamic light scattering, which results from the stochastic movement of the dispersed vesicles. The same setup used to measure vesicles’ electrophoretic mobility with a helium-neon laser beam at 633 nm was also used to evaluate ZP. PS, PDI, and ZP results were recorded in triplicate [58,59].

##### Determination of TCR Entrapment Efficiency (EE%)

The indirect method was used to determine the entrapment efficiency of TCR-Cubs. An aliquot (1 mL) of cubosomal dispersion was subjected to ultracentrifugation (Sigma 3-30 KS cooling ultracentrifuge, Sigma Laborzentrifugen GmbH, Osterode am Harz, Germany) for 1 h at 22,000 rpm and a temperature of 4 °C. TCR that was not trapped was quantified from the supernatant via HPLC, as detailed above, with UV detection at approximately 220 nm after a 1:10 dilution with methanol. The %EE results are presented as (mean ± SD) of three separate experiments [60,61]. The following equation (Equation (1)):

(1) $$\mathrm{EE}\%=\frac{Total\;TCR\;concentration-Concentration\;of\;unentrapped\;TCR}{Total\;TCR\;concentration}\times 100$$

#### 2.3.5. Statistical Optimization of TCR-Cubs

To optimize the independent variables statistically, the numerical desirability algorithms embedded in Design-Expert^®^ software were used, considering the constraints on the measured responses outlined in Table 1. To evaluate the model’s predictive accuracy, the proposed optimal formula was computed, and the target responses were subsequently reassessed. As a follow-up, a one-way ANOVA was used to test for a statistically significant difference (α = 0.05) between the measured response means and their predicted counterparts [37,62].

#### 2.3.6. Physicochemical Characterization of the Optimum Formula

##### Transmission Electron Microscopy (TEM)

A Transmission Electron Microscope (TEM) (Joel JEM 1400, JEOL Ltd., Tokyo, Japan) was used to examine the optimal TCR-Cubs vesicles. The cubosomal dispersion was diluted (1: 20) with double-distilled water and filtered to remove clumps. 2 drops of the dispersion were prepared and stained with 1% phosphotungstic acid. A sample was prepared by dipping a carbon-coated copper grid into the dispersion and air-drying it to form a thin film, which was later examined [63,64].

##### Fourier Transform Infrared Spectroscopy (FTIR) Studies

The different formulations were stored at −2 °C and then subjected to freeze-drying in a lyophilizer (Model: Novalyphe-NL 500; Savant Instruments Corp., Holbrook, NY, USA) operated at −45 °C and 7 × 10^−2^ mBAR for 24 h. Subsequently, the TCR, blank cubs, and the freeze-dried optimum TCR-cubs were studied using an FTIR spectrophotometer (Bruker Model 22, Bruker Corporation, Bremen, Germany) [65,66]. Briefly, each sample was accurately weighed to 5 mg, mixed with dry potassium bromide, and then the sample and KBr were compressed into a thin disk using the geometric dilution method. The final disk was scanned at room temperature (25 °C ± 2 °C) over the range 4000–500 cm^−1^ [67,68].

##### Crystallinity Examination via Differential Scanning Calorimetry (DSC)

Differential scanning calorimetry (DSC) analysis assessed crystallinity and potential interactions among TCR, Blank Cubs, and the freeze-dried optimal TCR-cubs to evaluate their thermotropic properties. Samples weighing 3 to 4 mg were heated in tightly sealed flat-bottomed aluminum pans over the temperature range 25 to 400 °C at a heating rate of 10 °C per minute under an inert nitrogen flow of 30 mL per minute. The differential scanning calorimeter (Shimadzu DSC 50; Shimadzu Corporation, Kyoto, Japan) was calibrated with purified indium (99.9%) and used to record the DSC thermogram [69,70].

##### Stability Study

The optimized TCR-Cubs formula was maintained at refrigerated (4 ± 1 °C) and ambient temperatures for 0, 3, and 6 months. To evaluate physical stability, pre-storage and post-storage values of PS, PDI, ZP, and EE% were compared [71].

#### 2.3.7. Fabrication of HPMC/PVP K90 Dissolving Microneedle Patches

The dissolving microneedle (MN) arrays were fabricated by a micromolding technique using 1 cm × 1 cm polydimethylsiloxane (PDMS) molds (Silicone Template ST-01; Micropoint Technologies Pte Ltd., Singapore). Each mold contained a 10 × 10 array of conical cavities with a height of 300 μm, a base width of 100 μm, and a tip diameter of 5 μm. Two water-soluble polymers, hydroxypropyl methylcellulose (HPMC) E50 and polyvinylpyrrolidone (PVP) K90, were used as the microneedle matrix. Separate stock solutions of HPMC E50 (7% *w*/*w*) and PVP K90 (40% *w*/*w*) were prepared in an ethanol–water mixture (8:2, *v*/*v*) under continuous stirring until complete dissolution. For each formulation, the HPMC and PVP stock solutions were mixed to achieve the desired HPMC: PVP K90 ratios shown in Table 3 (HPMC/PVP K90/MNs1: 20/80; HPMC/PVP K90/MNs2: 33/67; HPMC/PVP K90/MNs3: 50/50, % *w*/*w* of total polymer solids). The resulting polymer blend, containing the pre-dispersed freeze-dried tacrolimus-loaded cubosomes (TCR-Cubs), was carefully poured onto the PDMS molds. The filled molds were placed in centrifuge tubes, and the formulation was driven into the microneedle cavities by centrifugation at 6000 rpm for 2 h (MPW-352R, MPW MED. INSTRUMENTS, Warsaw, Poland). After centrifugation, the molds were removed and allowed to dry at ambient temperature for 24 h to ensure complete solvent evaporation and microneedle solidification [72,73]. Finally, the solidified HPMC/PVP K90 dissolving microneedle patches, each containing TCR-Cubs, were gently peeled off from the molds and stored in a desiccator until further characterization, as shown in Figure 1b.

#### 2.3.8. Characterization of HPMC/PVP K90 Dissolving Microneedles Loaded with Freeze-Dried TCR-Cubs Microneedle Patches

##### Drug Contents

Magnetic agitators comprising TCR-Cubs/HPMC-PVP/MNs were dissolved in distilled water with 2.5% Tween 80 and agitated at 300 revolutions per minute for one hour. The solution was diluted with methanol and sonicated for 5 min to ensure complete dissolution of TCR within the TCR-Cubs loaded HPMC/PVP-MNs. The validated HPLC method described in the previous section was used to perform this procedure. The drug content of the produced HPMC/PVP-MNs was then validated [74,75].

##### Mechanical Strength and Penetration Capability Test

The mechanical strength and penetration effectiveness of TCR-Cubs/HPMC-PVP-MNs were evaluated in this study. Eight layers of Parafilm M^®^ (Bemis Company, Inc., Neenah, WI, USA), a flexible thermoplastic sheet composed of an olefin-type substance that closely resembles the thickness and texture of human skin, were used to evaluate the different MNs. For 30 s, MNs were exposed to 32 N (3.2 kg), or the typical adult thumb pressure. Measurements were taken of the MNs’ height and form, as well as the quantity of holes in each Parafilm M^®^ layer [74,76]. The mechanical strength and penetrating capacity were computed using Equations (2) and (3).

(2) $$\%\;\mathrm{Height}\;\mathrm{reduction}=\frac{Initial\;height-Final\;height}{Initial\;height}\times 100\%$$

(3) $$\%\;\mathrm{Penetration}\;\mathrm{capability}=\frac{Number\;of\;holes}{Total\;needles}\times 100\%$$

##### Water Loss on Drying (LOD)

To determine the limit of detection (LOD), 0.5 g of MNs polymers was accurately weighed into a mold. The polymers were dried in a desiccator and weighed again after 48 h [58]. The data from these measurements were analyzed using the following equations:

(4) $$\%\mathrm{LOD}\;=\;\frac{Initial\;weight-Final\;weight}{Initial\;weight\;}\times 100$$

#### 2.3.9. Characterization of Optimized Microneedle

##### Scanning Electron Microscopy (SEM)

Scanning electron microscopy (SEM) is a technique that uses a focused electron beam to generate high-resolution images of surfaces. This approach facilitates the study of the topography and composition of materials at the micro- and nanoscale. The morphology of the TCR-Cubs/HPMC-PVP/MNs patch was studied with a scanning electron microscope (SU8010, Hitachi, Tokyo, Japan). The TCR patch was mounted on a microscope carriage, and imaging was performed at 6 kV with 150× magnification [77,78].

##### Fourier Transform Infrared (FTIR) Analysis

An FTIR spectrometer was used to record the room-temperature spectra of a TCR-Cubs/HPMC-PVP/MNs patch and its constituent parts between 4000 and 500 cm^−1^ (model 22, Bruker, Coventry, UK).

##### Differential Scanning Calorimetry (DSC)

The thermal properties, crystallinity, and interactions of the TCR-Cubs/HPMC-PVP/MNs patch were examined using differential scanning calorimetry (DSC-60; Shimadzu, Kyoto, Japan) at temperatures from 25 to 250 °C under nitrogen purge at 100 mL/min [79], as described previously.

##### Drug Release Studies

Comparative In Vitro Release Study

The in vitro release profiles of TCR-Cubs, TCR-Cubs/HPMC-PVP/MNs, and a TCR suspension (1 mg/mL) were compared using the dialysis bag technique. A Spectra Por^®^ dialysis membrane (12,000–14,000 Da) was conditioned overnight in the release medium. An accurately measured quantity of either TCR-Cubs, TCR-Cubs/HPMC-PVP/MNs, or TCR suspension, equivalent to 5 mg of TCR, was placed in the donor compartment of the dialysis bag. The bag was then sealed and placed in stoppered glass bottles containing 50 mL of pH 7.4 PBS with 20% methanol (80:20% *v*/*v*) as the release medium in the receptor compartment. The bottles were placed in a shaking water bath at 37 ± 0.5 °C and 60 strokes per minute (Unimax, IKA, Staufen, Germany). Samples of 1.5 mL were collected at 0, 0.5, 1, 2, 4, 6, 8, 12, and 24 h, and the release medium was replaced with fresh release medium to maintain sink conditions. The samples were analyzed by HPLC at 220 nm to determine the percentage of TCR released at each time point. The release profiles are expressed as the mean of three experimental replicates. Kinetic analysis of the in vitro release profiles was performed using the zero-order, first-order, Higuchi diffusion, and Korsmeyer–Peppas models. The coefficient of determination (R^2^) for each model was calculated, and the model with the highest R^2^ was used to describe the release kinetics of the optimized formula [80,81].

Corneal Ex Vivo Permeation Study

Corneal samples were obtained from male albino rabbits. The rabbits were anesthetized with a combination of ketamine (200 mg) and xylazine (20 mg) and then euthanized by decapitation. The corneas from the removed eyeballs were extracted and prepared by saline cleaning to avoid plications or folds that would compromise the integrity of the membrane prior to mounting. The transparent corneas used in permeation experiments were processed within 30 min of the animals’ death. The ex vivo permeation study of TCR was performed as three independent experiments using a Franz diffusion cell (Hanson Research, Chatsworth, LA, USA) with a donor and a receptor compartment. In the donor compartment, 1.5 mL of TCR-Cubs, TCR-Cubs/HPMC-PVP/MNs, or TCR suspension (5 mg) was added. At the same time, 25 mL of freshly prepared simulated tear fluid (pH 7.4) was added to the receptor compartment. A plastic membrane was used to hold the cornea in place, thereby maintaining a consistent diffusion surface area of 0.785 cm^2^. This membrane, which holds the cornea, was placed between the two compartments. The cells were kept at 37 ± 0.2 °C and stirred at 50 rpm using a magnetic stirrer. In the receptor compartment, aliquots (2 mL) were removed at the 0-, 1-, 2-, 4-, 6-, 8-, 10-, 12-, and 24 h time points, and fresh medium was added [82,83]. The samples were filtered through a 0.22 μm nylon filter, and the filtrates were compared with a blank of the release medium [84]. The cornea was placed in the medium with no active formula, and the blank samples were withdrawn at the same time as the active samples at the defined intervals. The TCR concentration was analyzed using HPLC at 220 nm. The total quantity of drug permeated (mg/cm^2^) over time (h) was graphed. Equations (5) and (6) are used to calculate the maximum flux (J_max_) at 24 h and the enhancement ratio (ER) [82,83]:

(5) $$J_{\max}=\;\frac{Amount\;of\;drug\;permeated}{Time\;\times \;area\;of\;membrane}$$

(6) $$\mathrm{ER}=\frac{Jmax\;of\;TCR-Cubs\;or\;TCR-Cubs/MNs}{Jmax\;of\;TCR\;suspension(Control)}$$

Corneal Hydration Level

In post-permeation analysis of ex vivo corneal permeation studies, corneal hydration levels were evaluated to assess corneal tissue damage. Assessing this parameter is necessary to quantify damage resulting from an ex vivo permeation study. Damage may stem from improper processing of the excised corneal tissues or from the hydrogel formulation, possibly indicating that the results from the ex vivo permeation study were obtained from intact corneal tissues. Each corneal tissue sample was exercised and prepared to remove any remaining surface contaminants, dried on filter paper to remove surface moisture, and weighed before drying (Wi). The corneal sample was then dried for 24 h at 50 °C and subsequently weighed again (Wf). Using Equation (7), the corneal hydration level (%HL) was calculated as follows [85,86]:

(7) $$\%\mathrm{HL}\;=\;[1-(\frac{Wi}{Wf})]\times 100$$

### 2.4. In Vivo Study

#### 2.4.1. Induction of Ocular Allergic Conjunctivitis

To investigate the ocular anti-inflammatory effects, the five groups of rabbits (*n* = 6) were divided into Group I, Negative Control (Normal Group), and another 4 groups sensitized intraperitoneally with ovalbumin (1.8 mg/kg) and aluminum hydroxide (90 mg/kg) (OVA/AH) at pH 7.4 on days 1, 8, and 15. Then, the sensitized rabbits were challenged with 100 μL of OVA eye drops (4 mg/mL in PBS) daily for a week [1,87,88]. Conjunctivitis was induced, and the rabbits were divided into the following five groups:

Group I: Negative Control (Normal Group);

Group II: Positive Control (Diseased Group);

Group III: Treatment with TCR Suspension;

Group IV: Treatment with TCR Cubs;

Group V: Treatment with TCR Cubs/HPMC-PVP/MNs Patch.

**Figure 2:**
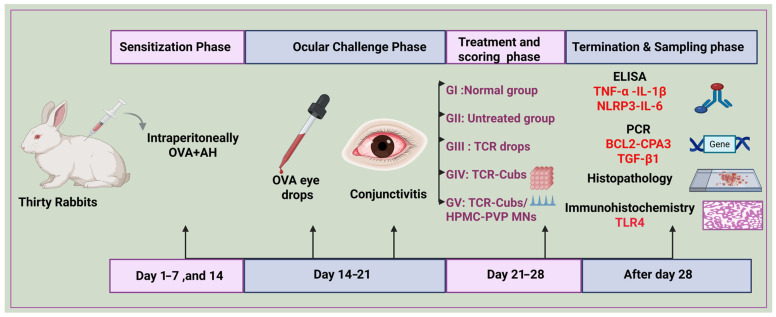
Experimental design of ovalbumin-induced allergic conjunctivitis model and evaluation of tacrolimus formulation therapy in rabbits.

The treatments were administered for a week. On day seven, the signs and symptoms of AC were scored across four parameters (discharge, redness, chemosis, and tearing). Each characteristic was scored from 0 (None) to 4+ (severe), with a maximum total score of 20+. The variations in symptom scores were plotted and presented graphically in Figure 2. The animals were euthanized at the end of the pharmacodynamic study, and the conjunctival and corneal tissues were dissected, cleaned, and fixed in 10% formalin for 1 day, then rinsed with saline for histopathological examination.

#### 2.4.2. Draize Test

Rabbits from each group (Group I: normal group, Group II: diseased group, Group III: treatment with TCR suspension, Group IV: TCR-Cubs, Group V: TCR-Cubs/HPMC-PVP/MNs) were administered specific volumes of the drug dispersion (0.1% *w/v* TCR) into the left eye cul-de-sac. The right eyes were used as negative controls. Rabbit eyes were examined using the slit lamp before, 1 h after, and 24 h after the last application. According to Draize test guidelines, eye irritation is evaluated as follows: (0–4) no irritation, (4–9) slight irritation, (9–13) moderate irritation, (13–19), and severe irritation [89,90].

#### 2.4.3. Enzyme-Linked Immunosorbent Assay (ELISA) for Inflammatory Markers

The levels of tumor necrosis factor-alpha (TNF-α), interleukin-1 beta (IL-1β), interleukin-6 (IL-6), and leucine-rich repeat and pyrin domain-containing protein 3 (NLRP3) were quantified in conjunctival/corneal tissue homogenates using commercially available sandwich ELISA kits (LifeSpan Biosciences, Inc., Seattle, WA, USA), according to the manufacturers’ instructions [75,91]. Briefly, tissue samples from all experimental groups were homogenized in ice-cold buffer containing protease inhibitors and centrifuged, and the clarified supernatants were used for analysis. Standards and samples were added to pre-coated plates, followed by biotinylated detection antibodies and streptavidin–HRP conjugate; color development was achieved with TMB substrate and stopped with 2 N H_2_SO_4_. Absorbance was measured at 450 nm using a microplate reader, and cytokine concentrations were calculated from standard curves and normalized to tissue weight (pg/g tissue) [92].

#### 2.4.4. Quantitative Real-Time Polymerase Chain Reaction (qRT-PCR)

Total RNA was isolated from ocular tissues of all groups using QIAzol reagent (Qiagen, Hilden, Germany), and RNA purity and concentration were determined spectrophotometrically (NanoDrop, Thermo Fisher Scientific, USA). One microgram of RNA was reverse-transcribed into cDNA using the SensiFAST™ cDNA Synthesis Kit (Bioline, London, UK), followed by amplification on an Applied Biosystems 7500 real-time PCR system (Applied Biosystems, Foster City, CA, USA) using HERA SYBR Green Master Mix (Willowfort, Nottingham, UK) under the following cycling conditions: 95 °C for 2 min, then 40 cycles of 95 °C for 10 s and 60 °C for 30 s. Each 20 µL reaction contained 10 µL SYBR Green mix, 2 µL cDNA, 1 µL of each primer, and 6 µL nuclease-free water. Relative mRNA expression of BCL2, CPA3, and TGF-β1 was calculated by the 2^−ΔΔCt^ method using GAPDH as the internal control [10,74]; primer sequences are listed in Table 4.

### 2.5. Histopathological Study

After the pharmacological trial, rabbits were euthanized by sodium phenobarbital injection in a marginal vein. Following enucleation, the eyeballs were fixed in 10% formalin. For the conjunctiva, after cutting, cleaning, and dehydrating in xylene, the tissues were embedded in paraffin after a series of dehydrating and clearing steps, and sectioned to 4–6 μm. The processed tissues were evaluated histologically by light microscopy after hematoxylin and eosin (H&E) staining and xylene deparaffinization [93,94,95].

### 2.6. Immunohistochemical (IHC) Examination of Toll-like Receptor 4 (TLR4)

IHC was performed to assess ocular surface inflammation by evaluating Toll-like receptor 4 (TLR4) expression in conjunctival tissue from the allergic conjunctivitis model. TLR4 is a pivotal pattern-recognition receptor involved in the initiation and modulation of allergic inflammation at the ocular surface, and its overexpression has been documented in both experimental and clinical allergic conjunctivitis. In this research, the paraffin-embedded eye sections from the different experimental groups were mounted on adhesive slides, deparaffinized, and rehydrated through graded alcohols and distilled water. Subsequently, heat-induced epitope retrieval was done, followed by incubation of the tissue sections with the primary anti-TLR4 antibody for 1 h at 25 °C. After a rinse, an HRP-labeled detection system was used according to the manufacturer’s protocol for the reaction product. Negative control slides were prepared by omitting the primary antibody. TLR4 immunopositivity was measured by counting the mean percent of stained areas in five random high-power fields for each group, giving a semi-quantitative measure of TLR4-mediated inflammatory activation in allergic conjunctivitis [93,94,96].

### 2.7. Statistical Analysis

Data analysis was done using GraphPad Prism 10 (GraphPad, San Diego, CA, USA), and the mean ± SD was calculated. One-way analysis of variance (ANOVA) with Tukey’s test was used for multiple groups, whereas the *t*-test was used for two groups. *p* < 0.05 was considered statistically significant.

## 3. Results and Discussion

### 3.1. Analysis of Box–Behnken Design

The Box–Behnken design was used to quantify how the GMO level (A), GMO: F127 ratio (B), and homogenization time (C) influence particle size (PS), PDI, zeta potential (ZP), and entrapment efficiency (EE%) of tacrolimus-loaded cubosomes, and to identify an optimized formulation (OF). The experimental runs covered low, medium, and high levels of each factor (Table 1), and the measured responses are summarized in Table 2.

### 3.2. The Effect of Formulation Variables on Entrapment Efficiency (EE%), Particle Size (PS), Polydispersity Index (PDI), and Zeta Potential (ZP)

#### 3.2.1. Entrapment Efficiency (EE%)

**Figure 3:**
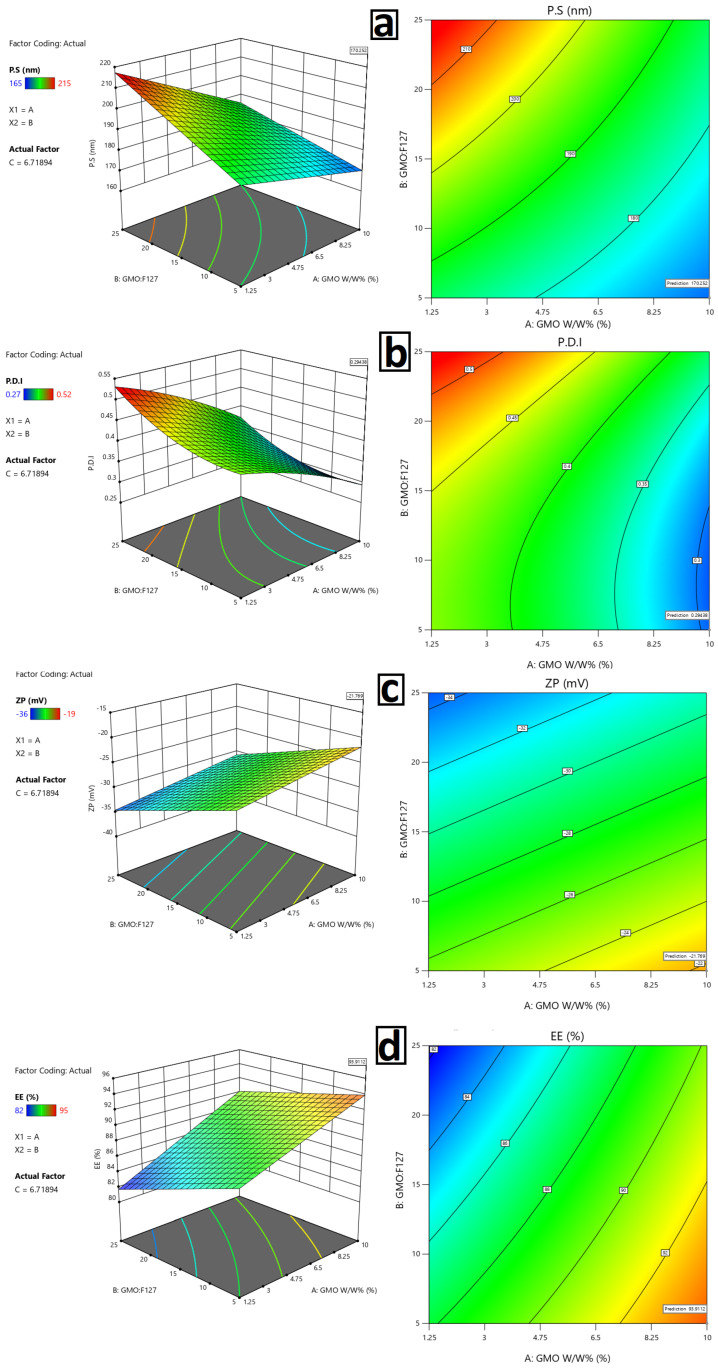
3D Response surface plot showing effect of A: GMO W/W%, B: GMO: F127., and C: homogenization time on (Y1) entrapment efficiency percentage (EE%) (**a**), (Y2) particle size (PS) (**b**), (Y3) polydispersity index (PDI) (**c**), and (Y4) zeta potential (ZP) (**d**).

Entrapment efficiency was primarily governed by the GMO content (A) and the GMO: F127 ratio (B). EE% varied from 82 ± 0.63% to 95 ± 0.83%. The highest EE values (≈95%) were obtained at high GMO (10%) and low F127 ratio (5) with either 5 or 10 min homogenization (runs 6 and 10), while the lowest EE (82–82.7%) was observed at low GMO (1.25%) with medium–high F127 (15–25) (runs 2 and 3) as shown in Table 2. This pattern indicates that increasing the lipid fraction provides a larger hydrophobic domain for tacrolimus accommodation, thus enhancing entrapment, while excessive surfactant can facilitate partition of the drug into the aqueous phase or solubilization outside the cubic matrix, reducing EE%. Similar trends have been reported for β-carotene, corticosteroids, and other hydrophobic drugs loaded into GMO cubosomes optimized by BBD, where lipid concentration positively and surfactant concentration negatively influenced EE% [97,98]. Homogenization time did not appear to be a significant factor in the final model for EE%, suggesting that, within the tested range (0–10 min), the fragmentation process did not substantially alter drug leakage. This is consistent with previous reports where processing conditions mainly affected PS/PDI, while EE% was determined predominantly by composition, as shown in Figure 3.

#### 3.2.2. The Effect of Formulation Variables on Particle Size Analysis of PDI

Particle size was significantly influenced by all three factors (A, B, and C). The smallest PS (165 ± 0.63 nm) was obtained at high GMO (10%), low GMO: F127 ratio, and medium homogenization time (5 min, run 6), while the largest PS (215 ± 0.54 nm) occurred at low GMO (1.25%), high F127, and medium homogenization (run 3) as shown in Table 2. In general, higher GMO levels tended to reduce PS within the tested range, particularly when combined with adequate homogenization, likely because a more robust cubic matrix forms that can be efficiently fragmented into smaller nanostructures. This aligns with studies stating that lipid content increases up to a certain point, encouraging the formation of compact cubic domains that subdivide into small cubosomes during high-energy processing. The ratio of GMO to F127 seemed to have a more complicated effect: at a constant GMO, increasing F127 from 5 to 15% often resulted in a decrease in particle size (GMO 10% and 5 min: PS 165 nm at ratio 5 vs. 181–182 nm at 15–25), while very high surfactant ratios in combination with low GMO (1.25%, ratios 15–25) produced relatively larger particles (199–215 nm, runs 2, 3, 7, and 9). This indicates that a stabilizer is helpful to a certain degree; beyond that, too much surfactant may trigger micellar solubilization or changes in viscosity that impede sufficient reduction in size [98,99,100]. This phenomenon was also noticed with the other BBD-optimized cubosomes and nano-lipid carriers, as seen in Figure 3.

Homogenization time (C) also plays a significant role in impacting PS: at a constant composition, moving from 0 to 5 min reduced particle size (e.g., GMO 10%, F127 15%: PS 169 nm at 0 min vs. 181 nm at 10 min, but 165 at 5 min with ratio 5), suggesting that moderate high-energy input enhances fragmentation of the bulk cubic gel. Extremely long or high-energy processing can sometimes lead to re-aggregation or structural rearrangements, which may account for the fact that PS did not consistently decrease over time across all compositions. Analogous non-linear processing time/pressure effects have been documented in the literature for cubosome and SLN systems. Values of PDI, which reflect the phenomena of declining PS, were also greatly influenced by A, B, and C. Formulations with high GMO, low F127, and moderate homogenization (runs 6 and OF) gave the lowest PDI of 0.27–0.29, while the highest PDI of 0.51–0.52 was recorded at low GMO and high F127 (runs 3 and 13). This illustrates that an optimized lipid–surfactant balance, along with sufficient homogenization, is key for narrow size distributions. This finding is consistent with previous optimizations of cubosomes aimed at achieving PDI < 0.3 for colloidal stability.

#### 3.2.3. Effect on Zeta Potential

Based on the two-factor interaction (2FI) model analysis, the only significant factor at B was the GMO: F127 ratio (B), while Zeta Potential (ZP) values ranged from 36 to −19 mV. Greater ZP (−35 to −36 mV at B = 25, Runs 3, 13, 15) ZP values were found at intermediate levels (5–10%) of GMO along with higher levels of F127, while lower ZP values (−19 to −24.8 mV) were found at lower F127 levels (B = 5) and high homogenization (Run 10, −19 mV) (Table 2). This shows that at high F127 concentrations, the surface composition and ionization of the GMO emulsion are modified, likely due to exposure or shielding of surface-active ions and adsorbed ions. A negative ZP is expected for GMO-based cubosomes due to the presence of free ionized fatty acids and adsorbed counterions. ZP values between −20 and −30 mV are generally accepted as adequate for electrostatic stabilization. ZP values more negative than −35 to −36 mV at higher F127 levels are indicative of increased electrostatic stabilization, consistent with increased dispersion stability. As for the other studies, they found that as the non-ionic stabilizer increased, the ZP values became more negative and aggregation was effectively suppressed. However, these compositions (high F127) tended to produce larger PS and higher PDI, highlighting the need to balance charge stabilization with size control when choosing the GMO: F127 ratio [97,99,100], as shown in Figure 3.

No viscoelastic gelation occurred as all formulations maintained F127 concentrations < 3% *w*/*w*, confirmed by flowable dispersions and SAXS cubic phase patterns. GMO co-solubilization prevented micelle packing required for gel formation, maintaining optimal colloidal stability [101]. The Box–Behnken design demonstrated significant (*p* < 0.001) quadratic effects for all three variables (R^2^ > 0.85). Elevated GMO concentrations (10% *w*/*w*) diminished PS from over 200 nm to 165–181 nm by generating resilient cubic phases conducive to ultrasonication fragmentation, and enhanced EE to 93–95% by enlarged hydrophobic domains facilitating tacrolimus partitioning. Low GMO:F127 ratios (5) reduced PDI (0.27–0.29) by producing limited F127 stabilized dispersions without micellar dilution of the cubic phase, whereas high ratios (25) elevated PDI (>0.36) due to excessive thickening of the polymeric corona. Ultrasonic homogenization demonstrated a parabolic response: 5–7 min were best for nano-sizing (particle size 165–210 nm), whereas shorter durations resulted in coarse dispersions, and extended durations induced re-aggregation due to heat effects. The improved formula (10% GMO, ratio 5, 7 min) attained a desirability of 0.92, validated empirically (predicted versus actual: PS 208 ± 6 nm vs. 210 ± 0.9 nm).

#### 3.2.4. Statistical Analysis of Formulation Variables on Particle Size (PS), Polydispersity Index (PDI), Zeta Potential (ZP), and Entrapment Efficiency (EE%)

Across all formulations, PS ranged from 165 to 215 nm, PDI from 0.27 to 0.52, ZP from −36 to −19 mV, and EE% from 82 to 95% (Table 5). The statistical analysis showed highly significant models for all responses: F-values were 88.82, 98.35, 21.23, and 13.16 for PS, PDI, ZP, and EE%, respectively, with *p* < 0.001 in all cases, indicating that the chosen factors explain most of the variability in the responses. The models for PS and PDI were quadratic with excellent R^2^ values (0.985 and 0.994) and high adjusted and predicted R^2^, confirming good fit and predictive capability. For ZP and EE%, two-factor interaction (2FI) models were adequate (R^2^ = 0.853 and 0.907), with acceptable adjusted and predicted R^2^, showing that interactions between factors, rather than higher-order terms, mainly govern these responses. Adequate precision values were >10 for all responses (28.855 for PS, 31.203 for PDI, 14.659 for ZP, 12.261 for EE%), indicating a sufficient signal-to-noise ratio and supporting the models’ reliability for navigating the design space. As shown in Table 5, the Box–Behnken optimizations of cubosomes and other lipid carriers, where R^2^ > 0.9 and non-significant lack-of-fit are typically reported for well-behaved designs.

#### 3.2.5. Optimization of Tacrolimus-Loaded Cubosomes (TCR-Cubs)

The numerical optimization was performed to maximize EE% and the magnitude of ZP (more negative), while minimizing PS and PDI. The optimized formula (OF) was predicted to be high in GMO (10%), low in F127 ratio (5), and intermediate in homogenization time (7 min). Experimentally, OF showed PS (210 ± 0.91 nm), PDI (0.29 ± 0.03), ZP (−21 ± 0.87 mV), and EE (93.3 ± 0.45%). These values are in close agreement with the model predictions and lie within the desirable window for stable, nanosized cubosomes with high drug loading. Compared to the other runs, OF offered a compromise between the smallest achievable size (165 nm) and the highest EE% (95%), while maintaining narrow PDI and sufficiently negative ZP for colloidal stability. Such trade-off solutions are commonly selected in desirability-based optimization of cubosomes, where extreme conditions that minimize PS may compromise EE, or vice versa. The final TCR-Cubs formulation thus exhibits physicochemical characteristics comparable to other optimized cubosomal systems reported for ocular and anticancer delivery (PS ~ 180–230 nm, PDI < 0.3, ZP around −20 to −30 mV, EE% > 80%), supporting its suitability as a nanocarrier for tacrolimus.

### 3.3. Physicochemical Characterization of the Optimum Formula

#### 3.3.1. Transmission Electron Microscopy (TEM)

**Figure 4:**
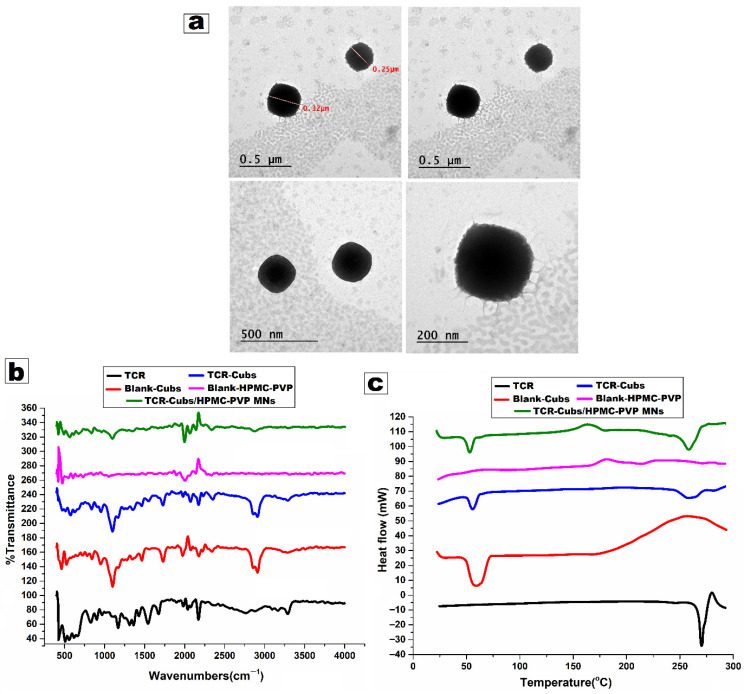
(**a**) TEM micrograph of optimized tacrolimus-loaded cubosomes (TCR-Cubs), (**b**) Fourier transform infrared spectroscopy, and (**c**) differential scanning calorimetry for pure tacrolimus (TCR), tacrolimus-loaded cubosomes (TCR-Cubs), b blank cubosomes (Blank-Cubs), blank HPMC-PVP/MNs (HPMC-PVP/MNs), and TCR-Cubs/HPMC-PVP/MNs.

The transmission electron micrographs (TEM) illustrated in Figure 4a show cubosomal nanoparticles loaded with tacrolimus that are nearly spherical in shape with smooth, dense cores and surrounding lighter coronas. These are consistent with a lipid cubic internal structure, which is stabilized by surfactant. The average diameter measured is between 250 and 320 nm (upper panels), which is consistent with DLS data for the optimized formulation (PS ~210 nm, PDI ~0.29), taking into consideration the fact that TEM measures dehydrated particle size, and a small number of larger particles are more prominent in the micrographs. The low PDI value confirms a monodisperse (uniform) population of nanocarriers for ocular delivery, where particles less than 300 nm are ideal for delivery systems to minimize irritation and avoid ocular obstruction. The bright/dark core/peripheral contrast indicates that tacrolimus is well entrapped in the hydrophobic GMO matrix, while the outer region may be a hydrated surfactant layer, consistent with the high entrapment efficiency (≈93–95%) relevant to the optimized formula. This structural organization is beneficial for ocular allergic conjunctivitis because it enables sustained release of tacrolimus from the lipid core while maintaining adequate colloidal stability in the tear film. Similar GMO/Poloxamer cubosomes have been reported to prolong precorneal residence and enhance corneal permeation of hydrophobic drugs, thereby reducing the dosing frequency compared with conventional solutions or ocuserts. The absence of pronounced aggregation or irregular shapes in the TEM images also supports the suitability of the chosen GMO/F127 ratio and homogenization time [102,103].

#### 3.3.2. Fourier Transform Infrared Spectroscopy (FTIR) Studies

Figure 4b shows FTIR spectra for pure tacrolimus, blank cubosomes, and TCR-Cubs; it shows characteristic bands of the macrolide structure, such as a strong carbonyl stretching band around ≈1730–1700 cm^−1^, N–H/amide bands in the 3300–3400 cm^−1^ region, and C–O–C/C–N vibrations in the 1000–1300 cm^−1^ region. These sharp, well-defined peaks are typical of the crystalline drug. The blank cubosomes display the expected GMO/Poloxamer bands (broad O–H stretching around 3400 cm^−1^, C–H stretching at ≈2920/2850 cm^−1^, ester C=O of GMO near 1730 cm^−1^, and C–O–C bands at ≈1100–1150 cm^−1^). After drug loading (TCR-Cubs), the main lipid–surfactant bands remain at essentially the same positions, while the distinct tacrolimus peaks are greatly attenuated or overlapped, rather than appearing as separate, sharp signals [104,105,106]. This masking of TCR characteristic peaks indicates that tacrolimus is molecularly dispersed or present in an amorphous/low-crystalline state within the GMO matrix rather than as a separate crystalline phase; the FTIR confirms successful encapsulation of tacrolimus in cubosomes, as shown in Figure 4b.

#### 3.3.3. Crystallinity Examination via Differential Scanning Calorimetry (DSC)

Figure 4c shows DSC thermograms for pure TCR (exhibits a sharp endothermic melting peak (T_m_) characteristic of crystalline tacrolimus, typically around ≈130–140 °C (exact value from your data). This confirms its initial crystalline nature. Blank cubosomes show broad endothermic events associated with melting and phase transitions of GMO and Poloxamer 407 (usually in the 40–70 °C region) and possibly additional small transitions at higher temperatures. The absence of any TCR-like sharp melting peak here confirms that these thermograms represent the excipient matrix only. In TCR-Cubs, the characteristic sharp TCR melting endotherm is absent or markedly reduced, and only the broader GMO/P407 transitions remain [99,107]. Pure tacrolimus displayed a distinct endothermic melting peak at around 130–140 °C, indicative of a crystalline substance. The peak was entirely absent in TCR-Cubs, corroborating molecular dispersion and amorphization within the GMO cubic matrix. Blank cubosomes exhibited extensive endothermic transitions at approximately 45 °C (GMO lamellar-to-cubic phase transition) and at 65 °C (F127 micelle rearrangement), while TCR-Cubs demonstrated analogous profiles with small alterations in onset attributed to drug-lipid packing interactions. These reversible thermodynamic phenomena confirm physical entrapment in the absence of a crystalline drug domain. This indicates that tacrolimus no longer exists in its crystalline form but is molecularly dispersed or amorphous within the cubic lipid matrix, which consists of the FTIR masking of drug bands, with high entrapment efficiency as shown in Figure 4c.

#### 3.3.4. Stability Study

The stability study in Table 6 shows that the optimized tacrolimus-loaded cubosomes (TCR-Cubs) maintain acceptable properties for 6 months. When stored in the fridge or at room temperature, TCR-Cubs show minimal changes to particle size, polydispersity, zeta potential, entrapment, and entrapment efficiency. Mean particle size (PS) of TCR-Cubs remained at the nanometric size for the entire 6-month study. At 24 h, the mean PS was 210–213 nanometers, and at 6 months, it was 189–185 nanometers. The modest decrease in PS indicates an insignificant reduction in the overall stability of the cubosomal nanostructures, suggesting no signs of aggregation. The PDI shows an increase in range from 0.29 to 0.31 to 0.45–0.49. This means that, over time, PDI showed a broad distribution. The PDI remains in an acceptable range for safety in drug delivery systems. Optimistic Dispersion (OD) shows PDI and PS with very low standard deviations in comparison to one another. This suggests that there was little to no change in the OD over time, resulting in stable TCR-Cubs that resist aggregation and sedimentation when stored in the fridge and at room temperature. The zeta potential (ZP) becomes increasingly negative over time, moving from approximately −21 mV at 24 h to between −35 and −41 mV by 6 months. An increase in the magnitude of the negative ZP value increases electrostatic repulsion between particles, thereby increasing colloidal stability and decreasing the likelihood of flocculation or coalescence. ZP values become more negative over time due to the slow re-organization of, or increased exposure of, ionizable moieties (e.g., charged surfactant or stabilizer groups) that become unearthed during the maturation of the cubosomal structures. Expressions for ZP values below −30 mV are associated with good kinetic stability in colloidal dispersions, indicating that they become more electrostatically stabilized over time rather than less. The entrapment efficiency (EE%) only decreased slightly over the storage duration of 6 months, from ~93–92% at 24 h to ~88–87% at 6 months which indicates that only very little leakage or degradation of tacrolimus occurred from the cubosomal matrix during that time which is expected for a highly lipophilic drug with a strong affinity for the lipid domains of cubosomes. The consistently high EE values in the study indicate that the cubic lipid phase continues to effectively encapsulate tacrolimus and that, during the evaluation period, no significant leaching or degradation of the drug occurred, regardless of storage conditions at refrigerated or ambient temperatures. The slightly lower EE at ambient temperature as compared to refrigeration suggests a very mild temperature-dependent effect on retention of the drug, but at a difference of approximately 1-2%, this is unlikely to be of any clinical significance, particularly given the storage stability demonstrated. Under both environmental conditions, storage at ambient temperature resulted in particle size and zeta potential (ZP) values comparable to or lower (more negative) than those observed under refrigerated conditions, while EE remained high in both conditions. This suggests that the formulation is structurally stable enough to be stored at room temperature without significant loss of quality attributes. This is also beneficial for clinical applications, as it eliminates the need for a refrigerator in stable environments. The stability results confirm that the formulation incorporates cyclodextrin and cubosomes (TCR-Cubs), which preserve its nanoscale size, narrow size distribution, and negative zeta potential, and maintain high levels of encapsulated tacrolimus over 6 months of evaluation, regardless of storage conditions (refrigerated or ambient). The lack of obvious particle development or significant drug depletion confirms that the cubosomal system is physically and chemically stable, affirming its validity as a long-term carrier for tacrolimus in upcoming microneedle loading and ocular delivery research

### 3.4. Characterization of Microneedle Patches

#### 3.4.1. Drug Content

**Figure 5:**
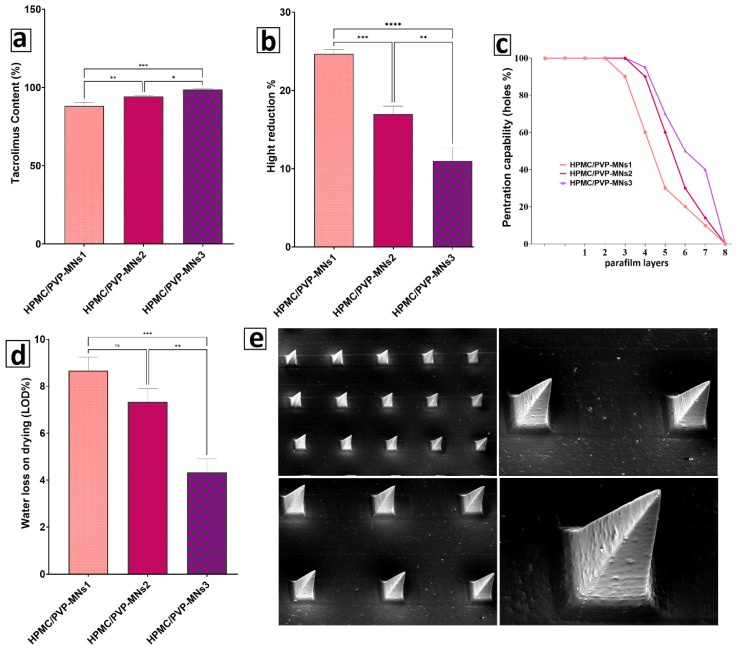
(**a**) Drug content (%) of tacrolimus (TCR) incorporated within TCR-Cubs loaded with HPMC-PVP/MNs, (**b**) percentage height reduction in HPMC-PVP/MNs (HPMC-PVP/MNs1–3), (**c**) penetration capability (%) of HPMC-PVP/MNs1–3, (**d**) water loss on drying (LOD, %) of HPMC-PVP/MNs formulations, and (**e**) scanning electron microscopy (SEM) images of optimized TCR-Cubs/HPMC-PVP/MNs 3; 100×, 200×, 350×, and 900×. Data are presented as mean ± SD (*n* = 3). Statistical significance was analyzed using one-way ANOVA followed by post hoc comparisons; * *p* < 0.05, ** *p* < 0.01, *** *p* < 0.001, and **** *p* < 0.0001.

Figure 5a, drug content and dose uniformity: proper, uniform drug loading across MN arrays promotes consistent dosing and predictable therapeutic outcomes. The tacrolimus content (%) in Figure 5a shows that all formulations contain a high percentage of the theoretical TCR dose, indicating that cubosomal dispersion survives the casting and drying processes with little drug loss or degradation. The relatively small SD bars show good batch-to-batch and needle-to-needle consistency, which is particularly important when using microneedles as “solid dosage forms” for localized therapy. However, the significant differences among HPMC-PVP/MNs1–3 imply that polymer composition subtly modulates drug distribution. Regarding MNs1, cases involving more water or less polymer may indicate that the cubosomes undergo phase separation or migration during drying, leading to less or more variable drug content. On the other hand, MNs3, which has a higher solid content and viscosity, appears to stabilize the TCR-Cubs more strongly within the casting solution, which reduces sedimentation and, therefore, increases the homogeneity of the tacrolimus loading. This strengthens the rationale for choosing MNs3 as it couples good mechanics with reliable dose delivery.

#### 3.4.2. Mechanical Strength and Penetration Capability Test

Figure 5b, height reduction (mechanical strength): the percentage reduction in height post-application is an indirect indicator of the mechanical robustness of the MNs: a greater reduction suggests the needles are soft and prone to bending or buckling, while a smaller reduction suggests sufficient stiffness to penetrate the skin without causing significant deformation. The literature on polymeric MNs suggests that a minimum fracture force coupled with minimal height loss is critical to reliably breach the stratum corneum. In the results, HPMC-PVP/MNs1 shows the greatest height reduction, suggesting that the needles may be undergoing excessive plastic deformation and are unlikely to achieve the desired depth within the viable epidermis. In contrast, HPMC-PVP/MNs3 exhibits a significantly greater reduction in height, suggesting a more resilient structure. This is likely due to the increased solid content and the improved HPMC:PVP ratio, which promotes the formation of a denser glassy network upon drying [108,109,110]. The statistically significant differences indicate that these changes in polymer composition affect mechanical functionality and are not due to random variation.

Figure 5c Penetration capability (%) of optimized HPMC-PVP/MNs1–3; the penetration capability, determined from layered parafilm, has long been accepted as an empirical measure for skin insertion efficiency. Many holes across multiple levels show that almost all the needles in the array can puncture the barrier to a clinically relevant depth and under a clinically applicable force. The results show that HPMC-PVP/MNs3 demonstrates the best penetration capabilities, particularly at deeper levels of the parafilm, whereas MNs1 shows a marked decrease in penetration with depth. This behavior aligns perfectly with the height-reduction results: stiffer MNs (MN3) with less deformation are better able to withstand the compressive forces of insertion and puncture the material, while softer MN1 are more likely to bend and “skive” rather than penetrate the surface [111,112]. For TCR delivery, efficient penetration is vital, as needles must completely penetrate the stratum corneum to reach the viable epidermis/dermis, allowing tacrolimus to be released into microcirculation. Although MNs3 has great penetration ability, which means more functional needles, hence, better certainty of drug delivery.

Figure 5d Water loss on drying (LOD, %) of HPMC-PVP/MNs formulations.

Residual moisture in polymeric microneedles influences the devices’ physical stability, brittleness, and microbial susceptibility. MNs1 has the highest loss on drying (LOD) among the HPMC-PVP MNs, indicating a more plastic and hydrated matrix. Although moisture can impart flexibility to the matrix, excessive moisture acts as a plasticizer, reducing Tg and overall mechanical toughness. This explains the greater reduction in MN height and the lower MNs1 penetration. Elevated water content increases the chance of hydrolytic drug degradation and microbial growth during storage. On the other hand, MNs3 exhibits a much lower LOD; it was more thoroughly dried, resulting in less residual water. This helps create a more rigid, glassy matrix with a higher Tg, thereby providing much greater mechanical strength and stability. In practice, the lower moisture content in MNs3 leads to less stickiness, greater ease of manipulation during packaging, and the potential for greater shelf stability without the need for rigorous storage conditions.

### 3.5. Characterization of Optimized Microneedle

#### 3.5.1. Scanning Electron Microscopy (SEM)

Figure 5e shows SEM images of TCR-Cubs/HPMC-PVP/MNs3, which show arrays of sharp pyramidal microneedles that are evenly spaced and of uniform height. Smooth, defect-free surfaces and pointed-tip geometry all help reduce insertion force and pain and create microchannels of uniform, precise dimensions in the epidermis. The absence of cracks, voids, or tip blunting indicates that the cubosomes and the drying process did not weaken the polymeric matrix. Furthermore, the uniform distribution of TCR-Cubs in microneedles is evidence that, in contrast to aggregation or sedimentation at the base of the microneedles, the TCR-Cubs were homogeneously distributed throughout the microneedles. This, along with the drug-content data, substantiates that a significant fraction of the microneedles may be cubosomes and will be inserted into the living layers of the skin, away from the superficial epidermis [1,37]. The micrographs show that optimizing the physicochemical parameters (polymer ratio, solid content, and drying conditions) is sufficient to produce a microneedle array that meets the specifications for mechanical and functional properties, enabling effective subcutaneous delivery of TCRs.

#### 3.5.2. Fourier Transform Infrared (FTIR) Analysis

Figure 4b displays FTIR spectra for Blank HPMC/PVP and TCR-Cubs/HPMC-PVP MNs; The HPMC/PVP spectrum has a predominant O-H stretch for the polymer around 3200–3500 cm^−1^, C-H stretch around 2900 cm^−1^, C=O of PVP’s lactam around 1650–1680 cm^−1^, and C-O-C bands around 1050–1150 cm^−1^. In the TCR-Cubs/HPMC–PVP microneedles, these polymer bands are retained with only minor broadening; no new peaks or major shifts in the tacrolimus or GMO carbonyl bands are observed. The absence of new absorption bands suggests no covalent interaction or chemical incompatibility between TCR, cubosomal lipids, and the HPMC/PVP matrix, supporting physical encapsulation and good compatibility.

#### 3.5.3. Differential Scanning Calorimetry (DSC)

Figure 4c shows DSC thermograms for Blank HPMC/PVP and TCR-Cubs/HPMC–PVP MNs. It also displays polymer-related thermal events: a glass transition or broad relaxation in the ≈70–120 °C range and possibly a broad endotherm due to moisture loss. In TCR-Cubs/HPMC–PVP MNs, the thermogram resembles that of the blank polymer matrix with slightly shifted/broadened transitions, but no distinct tacrolimus melting peak is visible. The disappearance of the TCR T_m_ in the microneedle thermogram confirms that the drug remains in an amorphous or molecularly dispersed state after incorporation into the HPMC/PVP microneedles and that there is no recrystallization during processing. Overall, the FTIR peak preservation (no new bands) and DSC disappearance of the tacrolimus melting peak together demonstrate that tacrolimus is stably encapsulated in the cubosomes and then in the microneedle matrix in a non-crystalline state, with good compatibility among TCR, GMO/P407, and HPMC/PVP, which is favorable for enhanced solubility and controlled release in the final formulation. Figure 4b (FTIR) and Figure 4c (DSC) together confirm successful encapsulation of tacrolimus in cubosomes and subsequent incorporation into the HPMC/PVP microneedles, with reduced crystallinity and no new interaction peaks, indicating compatibility of all components.

#### 3.5.4. Drug Release Studies

##### In Vitro Drug Release Study

**Figure 6:**
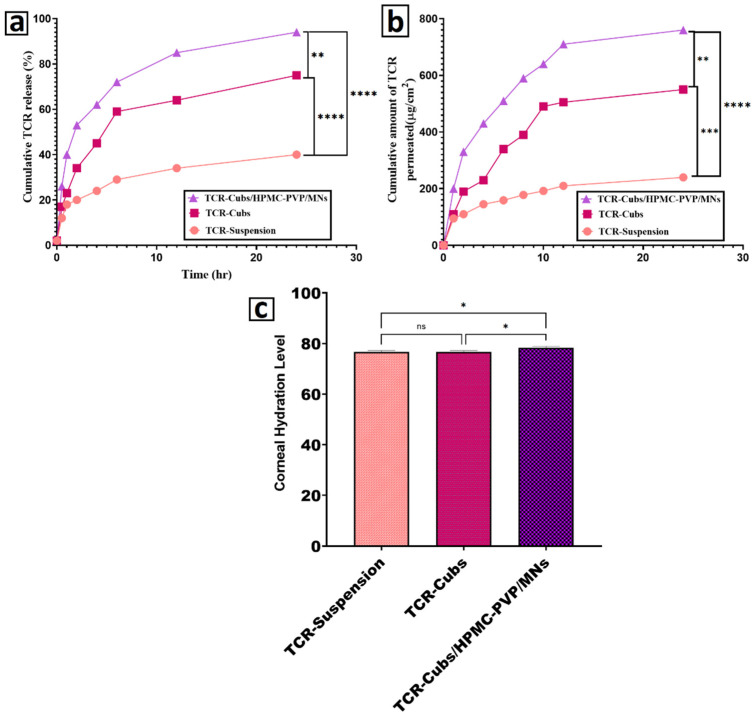
In vitro drug release and ex vivo permeation profiles of TCR formulations. (**a**) Cumulative in vitro release (%) of tacrolimus from TCR suspension, TCR-loaded cubosomes (TCR-Cubs), and TCR-Cubs-loaded HPMC-PVP K90 microneedles (TCR-Cubs/HPMC-PVP MNs). (**b**) Ex vivo cumulative permeated amount of TCR suspension, TCR-loaded cubosomes (TCR-Cubs), and TCR-Cubs-loaded HPMC-PVP K90 microneedles (TCR-Cubs/HPMC-PVP MNs) under the same experimental conditions, and (**c**) corneal hydration level for the group treated by TCR suspension, TCR-loaded cubosomes (TCR-Cubs), and TCR-Cubs-loaded HPMC-PVP K90 microneedles (TCR-Cubs/HPMC-PVP MNs). Data are presented as mean ± SD (*n* = 3). Statistical significance was analyzed using one-way ANOVA followed by post hoc comparisons; ns = not significant, * *p* < 0.05, ** *p* < 0.01, *** *p* < 0.001, and **** *p* < 0.0001.

Cumulative release increases in the following order: TCR-Suspension < TCR-Cubs < TCR-Cubs/HPMC-PVP MNs (Figure 6a). Under sink conditions, the suspension is only expected to release approximately 40% of tacrolimus at 24 h. This is due to tacrolimus’s high lipophilicity and poor water solubility. The drug is known to be dissolution-limited because it must first dissolve from the crystalline particles before diffusing through the dialysis membrane. Slow and incomplete release is paralleled in the literature with that of tacrolimus ointments and simple suspensions. Release is improved to approximately 75% at 24 h with tacrolimus encapsulated in cubosomes, with a more rapid initial phase. This is because, in this scenario, tacrolimus is molecularly dispersed in the lipid cubic matrix, which provides a larger internal surface area, meaning that only diffusion through the hydrated lipid channels coupled with the dialysis membrane is release-limiting. Release of hydrophobic drugs is known to be improved relative to suspensions, due to increased apparent solubility and a reduced diffusion path when using lipid nanoparticles and cubosomes [113,114]. A pronounced early burst, as well as the rapid and maximal release (≈95% at 24 h), is seen with the TCR-Cubs/HPMC-PVP microneedles. As the medium of release is introduced into the hydrophilic HPMC/PVP matrix, it instantly begins to dissolve, releasing the pre-distributed cubosomes into the surrounding fluid. This process completely sidesteps the initial wetting barrier that cubosomes also face within the dialysis bag. Other drugs that utilize dissolving microneedle systems have also shown the same behavior: rapid matrix erosion followed by diffusion-controlled release due to the embedded nanoparticles. Thus, the higher cumulative release attributed to the microneedle formulation is due to the rapid release of the carrier and the good diffusional characteristics of the cubosomes.

Tacrolimus release from the three formulations was also conducted in vitro and was followed by various kinetic models (zero-order, first-order, Higuchi, Korsmeyer–Peppas) to determine the underlying release mechanism, as shown in Table 7. Among the models, the tacrolimus suspension (Free TCR) was best fit by the Korsmeyer–Peppas model (R^2^ = 0.96) and the Higuchi model (R^2^ = 0.93). The zero-order and first-order models showed lower R^2^ values for their fits (0.72 and 0.78, respectively). The exponent for release (n ≈ 0.32) indicates a diffusion-controlled release mechanism, characteristic of Fickian diffusion. This suggests that the release of the drug from the suspension is primarily dependent on the diffusion of dissolved tacrolimus from the surfaces of slowly dissolving, poorly soluble drug particles, consistent with the behavior of fine particles. For the optimized tacrolimus-loaded cubosomes (TCR-Cubs), the Korsmeyer–Peppas model once more yielded the best fit (R^2^ = 0.99), with Higuchi R^2^ 0.92 and zero- and first-order R^2^ being lower (0.71 and 0.85, respectively). The Peppas release exponent for TCR-Cubs (n ≈ 0.50) is at the boundary between purely Fickian and anomalous transport, indicating that predominantly diffusive processes = transport are responsible for drug release through the bicontinuous lipid–aqueous channels of the cubosomal matrix, with a secondary role from processes that relax or reorganize the nanostructured lipid phase as it hydrates. This is consistent with previous reports describing mixed processes of diffusion and relaxation from lipid-based and other structured nanocarriers. In comparison, tacrolimus-loaded cubosome microneedles (TCR-Cubs/HPMC-PVP K90-MNs) recorded the highest R^2^ (0.94) for the first-order model, and Korsmeyer–Peppas and Higuchi models were slightly lower (0.97 and 0.89, respectively) while the zero-order fit was the worst (R^2^ = 0.65). The Peppas exponent for the microneedles (n ≈ 0.39) is in the Fickian diffusion regime, but the first-order fit, being the highest, indicates that after the microneedle matrix is hydrated and begins to dissolve/erode, the release of tacrolimus is not strictly governed by time but by the amount of tacrolimus in the polymeric network. This shows that there is diffusion-controlled release from the dissolving HPMC–PVP K90 matrix, and as the matrix dissolves, further drug-loaded cubosomes are uncovered, which maintains a concentration-dependent release profile. Overall, the data show that tacrolimus-loaded cubosomes embedded in the HPMC–PVP K90 microneedle matrix alter the release kinetics in contrast to the free drug and to cubosomes alone, moving the system from being predominantly Peppas-type diffusion in a lipid nanostructure to first-order, diffusion-controlled release governed by hydration and dissolution of the polymeric microneedle matrix. This kinetic transition justifies the use of cubosome-loaded microneedles for controlled drug release coupled with rapid dissolution of microneedle tips following skin insertion.

Ex Vivo Permeation Studies

The ex vivo permeation curves (Figure 6b) mirror the release data: cumulative amount permeated and Jmax at 24 h follow suspension << cubosomes < cubosomes/MNs. Tacrolimus suspension shows the lowest flux because only the small, dissolved fraction in the donor chamber can cross the corneal epithelium; this is consistent with earlier work where tacrolimus suspensions or ointments produced modest corneal levels. By contrast, cubosomes significantly enhance permeation. First, they keep tacrolimus in a solubilized state in the donor phase; second, their nanometric size and lipid composition promote close interaction with the corneal surface and possibly transient modulation of tight junctions, which increases transcellular and paracellular transport. Studies on tacrolimus nanocapsules and nanostructured lipid carriers have shown several-fold increases in corneal penetration compared with conventional formulations. The cubosome-loaded microneedles provide the highest permeation. In the ex vivo Franz cell model, the microneedles dissolve to form cubosomes, which are released very close to the epithelial surface; in vivo, microneedles dissolve to form cubosomes, which create microchannels in the superficial epithelium. Even in the ex vivo setup, this closer placement to the endpoint shortens the diffusion distance from the donor to the cornea and increases the effective concentration gradient, producing the greatest J_max_ and cumulative permeation. Similar reports are available for dissolving microneedles delivering antiviral or anti-glaucoma drugs, in which the flux through ocular or skin tissues is considerably greater than that observed with topical nanoparticles. The results from the in vitro and ex vivo studies are in line with pharmacotechnical expectations: the more sophisticated the delivery system (suspension, cubosomes, and cubosomes/MNs), the greater its ability to overcome the dissolution and barrier limitations of tacrolimus and to push the drug toward the receptor side.

Corneal Hydration and Safety

The normal level of hydration in the rabbit cornea is between 75 and 80%. Values above this suggest the presence of stromal edema and a loss of barrier function when corneal hydration exceeds 83–85%. The three formulations tested produced hydration values within this physiological range (Figure 6c). There is no significant difference between suspension and cubosomes, and only a small, statistically significant increase in the microneedle group, which remains below the edema threshold. This indicates that neither the cubosomal lipids nor the microneedle insertion/dissolution process caused meaningful corneal swelling or structural compromise during the experiment. Similar safety has been reported for other ocular nanocarriers and dissolving microneedles, which typically do not raise hydration above normal levels when appropriately formulated [115,116].

### 3.6. In Vivo Study

#### 3.6.1. Draize Test

**Figure 7:**
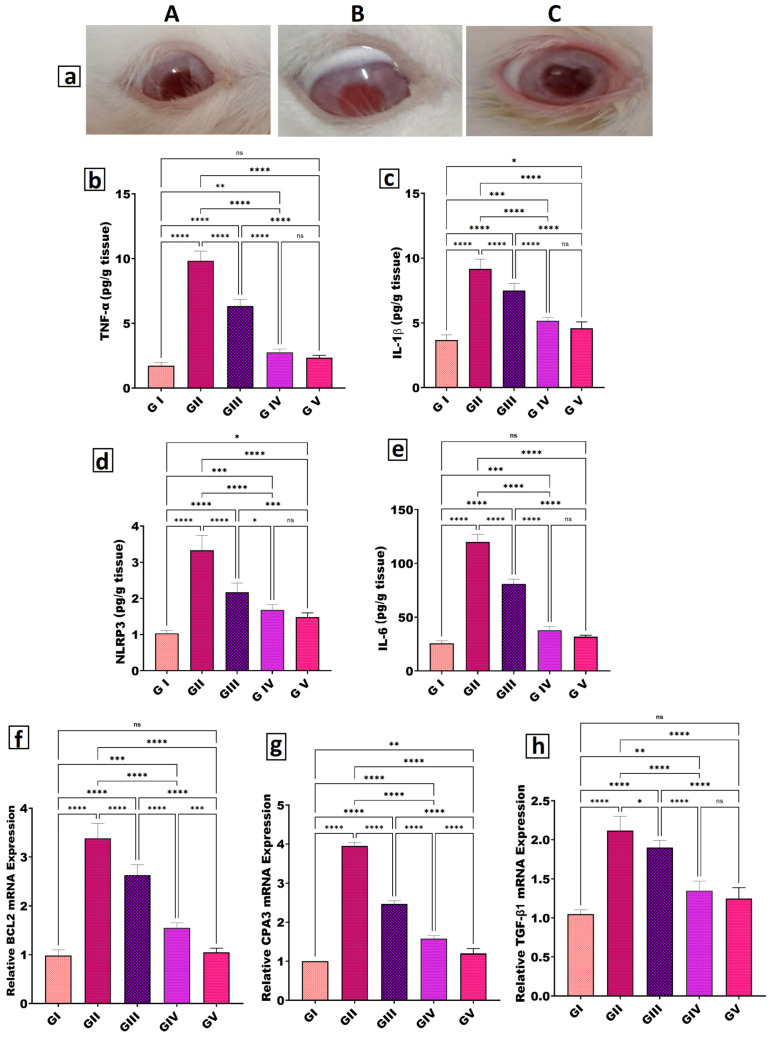
(**a**) Draize test, A: untreated allergic, B: tacrolimus suspension, C: tacrolimus cubo-somes/HPMC–PVP microneedles; (**b**–**e**) effects of different treatments on inflammatory biomarkers measured by ELISA: TNF-α, (**b**) IL-1β, (**c**) NLRP3 (**d**), and (**e**) IL-6. qRT-PCR analysis demonstrating the relative transcription levels of BCL2 (**f**), CPA3 (**g**), and TGF-β1 (**h**). Data are expressed as mean ± SD (*n* = 10).* *p* < 0.05, ** *p* < 0.01, *** *p* < 0.001, **** *p* < 0.0001, and ns (not significant) versus the control group. **Abbreviations**: Group I: Normal Group; Group II: Positive Control (Diseased Group); Group III: Treatment with TCR Suspension; Group IV: Treatment with TCR Cubs; and Group V: Treatment with TCR Cubs/HPMC-PVP/MNs Patch. Data were expressed as mean ± SD, *n* = 6.

The Draize test, as shown in Figure 7h. shows severe conjunctival hyperemia and chemosis in the untreated allergic eyes (panel A), partial improvement with tacrolimus suspension (panel B), and almost normal appearance after treatment with tacrolimus cubosomes/HPMC–PVP microneedles (panel C). This clinical picture parallels the biochemical and molecular findings and aligns with clinical studies showing that topical tacrolimus significantly improves redness, itching, and papillary reaction in severe allergic conjunctivitis [117,118,119].

#### 3.6.2. ELISA (Enzyme-Linked Immunosorbent Assay)

In the diseased group (GII), the concentrations of TNF-α, IL-1β, IL-6, and NLRP3 proteins are markedly higher than those in the normal group (GI), indicating activation of conjunctival NF-κB-associated cytokine and NLRP3 inflammasome signaling. Similar increases in these markers are reported in ocular surface inflammation and allergy, where NLRP3 activation promotes IL-1β and IL-6 production, thereby disrupting the barrier and inducing leukocyte infiltration. tacrolimus suspension (GIII) reduces cytokine production, particularly compared with GII; however, cytokine levels remained significantly elevated, indicating limited control of inflammation. Tacrolimus suspension (GIII) reduces cytokine levels compared with GII; however, cytokine levels remain significantly elevated, suggesting limited control of inflammation. The traditional topical treatment with tacrolimus has been shown to improve signs of allergic eye disease but has been limited by the drug’s availability in the target tissues of the cornea and conjunctiva. Tacrolimus-loaded cubosomes (GIV) have been shown to reduce TNF-α, IL-1β, IL-6, and NLRP3 to a greater extent than the suspension, indicating increased drug penetration and sustained drug exposure from the nanocarrier [120,121,122]. Formulations of nanoparticles with Tacrolimus in the eye have been shown to have better macrophage and neutrophil suppression and to provide superior histological protection in keratitis and uveitis models than other formulations of simple eye ointment or other solutions of tacrolimus. The GV cubosomes/HPMC-PVP microneedle patch has the lowest cytokine levels, which approach those in the normal control group and are often not significantly different from GI, indicating that the patch likely suppresses nearly the entire local inflammatory process. This indicates that the innovative approach of combining cubosomes with dissolving microneedles enhances the transconjunctival delivery of tacrolimus and is therefore likely to be more effective in inhibiting calcineurin–NFAT signaling and suppressing NF-κB-mediated cytokine transcription.

#### 3.6.3. Effect of the RNA Extraction and Real-Time PCR on Transcription Levels

At the cellular level, GII shows significant mRNA upregulation of BCL2 and CPA3, which are associated with increased survival of inflammatory cells and increased mast cell burden, respectively. BCL2 is associated with prolonged survival of eosinophils and lymphocytes in allergic tissues, and CPA3 is a mast cell protease associated with mast cell degranulation and is a hallmark of Th2-driven eosinophilic inflammation. Tacrolimus suspension only slightly decreases these transcripts, whereas cubosomes and cubosome–MNs significantly downregulate BCL2 and CPA3, bringing them close to baseline, reflecting decreased activation of mast cells/eosinophils and reduced persistence of inflammatory cells. This correlates with the established capacity of tacrolimus to attenuate mast cell mediator release and T cell-dependent survival signals in allergic diseases. TGF-β1 mRNA is also elevated in the diseased group, reflecting a fibrotic and remodeling-prone environment commonly seen in chronic allergic conjunctivitis and other eosinophilic disorders [123,124]. All tacrolimus treatments reduce TGF-β1 expression, with the cubosome–MN group showing the greatest decrease, suggesting a capacity to limit conjunctival remodeling and scarring in the long term. Ovalbumin-induced allergic conjunctivitis elicits a strong inflammatory and mast cell/eosinophils response in conjunctival tissues, and tacrolimus, notably in the cubosome–microneedle system, substantially inhibits these pathways [124,125]. The collective data from the ELISA, qPCR, and Draize assays suggest that tacrolimus delivered through cubosomes and, more importantly, through cubosome-loaded dissolving microneedles, demonstrate significantly greater anti-inflammatory and mast-cell-modulating effects than the suspension formulation. This results in the normalization of cytokine and gene-expression profiles, which correlates with the observed clinical improvement in the allergic rabbit eye model.

### 3.7. Histopathological Analysis

**Figure 8:**
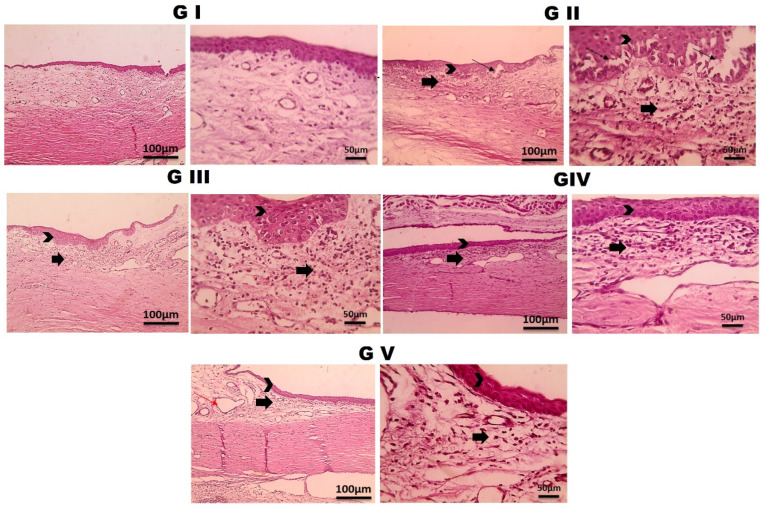
Microscopic histopathologic examination of the conjunctiva in allergic conjunctivitis model rabbits after topical application of different tacrolimus formulations. Meanwhile, the conjunctiva from the Diseased Group (GII) was subjected to infection, showing markedly increased thickness of the epithelial layer (arrowheads), inter-epithelial edema (thin black arrows), and many inflammatory cells infiltrating the subconjunctival space (thick black arrows). Conjunctiva from treated Group III showing decreased thickness of the epithelial layer (arrowheads), with many inflammatory cells infiltrating the subconjunctival space (thick black arrows). Conjunctiva from treated Group IV showing a greater decrease in thickness of the epithelial layer (arrowheads), and some inflammatory cells infiltrating the subconjunctival space (thick black arrows). Conjunctiva from treated Group V showing normal thickness of the epithelial layer (arrowheads), edema with dilated lymphatics (red arrow), and a few inflammatory cells infiltrating the subconjunctival space (thick black arrows). Low magnifications ×: 100 bar 100 and high magnifications ×: 400 bar 50. **Abbreviations**: Group I: Normal Group; Group II: Positive Control (Diseased Group); Group III: Treatment with TCR Suspension; Group IV: Treatment with TCR Cubs; and Group V: Treatment with the TCR Cubs/HPMC-PVP/MNs Patch. Data were expressed as mean ± SD, *n* = 4.

Histopathology illustrated in Figure 8 indicates that the ovalbumin-induced allergic conjunctivitis model is characterized by conjunctival inflammation, and that tacrolimus, particularly in the cubosome-microneedle system, ameliorated these alterations. Healthy conjunctiva (GI) has a thin and regularly organized epithelium that is not edematous; the basement membrane is intact, and there is only a sparse population of cells in the substantia propria. This is consistent with descriptions of healthy rabbit conjunctiva in the literature. On the other hand, the diseased group (GII) exhibits allergic conjunctivitis with pronounced epithelial thickening and detectable papillary change (arrowheads). Significant inter-epithelial edema is present as well as a dense infiltrate of inflammatory cells in the subconjunctival stroma (thick arrows). Epithelial hyperplasia and eosinophil/mast cell infiltration in the stroma have been previously described in both ovalbumin-induced models of ocular allergy and in vernal keratoconjunctivitis/atopic keratoconjunctivitis (VKC/AKC) in humans. Tacrolimus suspension treatment (GIII) results in some reduction in epithelial thickening compared to GII; however, many inflammatory cells remain in the substantia propria, suggesting only incomplete control of the allergic response [126,127]. This observation agrees with clinical findings that conventional topical tacrolimus improves disease signs but is limited by the poor ocular bioavailability of hydrophobic drugs. In GIV, where tacrolimus-loaded cubosomes were used, the epithelium is further normalized, and there is stromal infiltration that is clearly less intense than in GIII, which corroborates the greater local delivery and anti-inflammatory action attributed to the nanocarrier-based tacrolimus. This is consistent with previous studies showing that tacrolimus nanoparticles or nanocapsules provide greater penetration and histological protection than standard eye-drop formulations. The cubosomes/HPMC–PVP microneedle patch (GV) is associated with almost normal epithelial thickness with only mild edema (dilated lymphatics, red arrows) and polyvascularization (thick arrows), thus signaling almost complete allergic damage and its repair. This indicates that tacrolimus cubosomes with dissolving microneedles optimally enhance local drug delivery to the conjunctival and peri-ocular tissues, leading to greater suppression of T-cell- and mast-cell-mediated inflammation than when used in combination with either suspension or cubosomes alone [128,129]. This is also the case in the advanced delivery of tacrolimus formulations compared to standard ones in the corneal graft rejection and uveitis models, where nanocarriers reduced the histological presence of inflammatory cells and tissue thickening.

### 3.8. Immunohistochemical (IHC) Examination of Toll-like Receptor 4 (TLR4)

**Figure 9:**
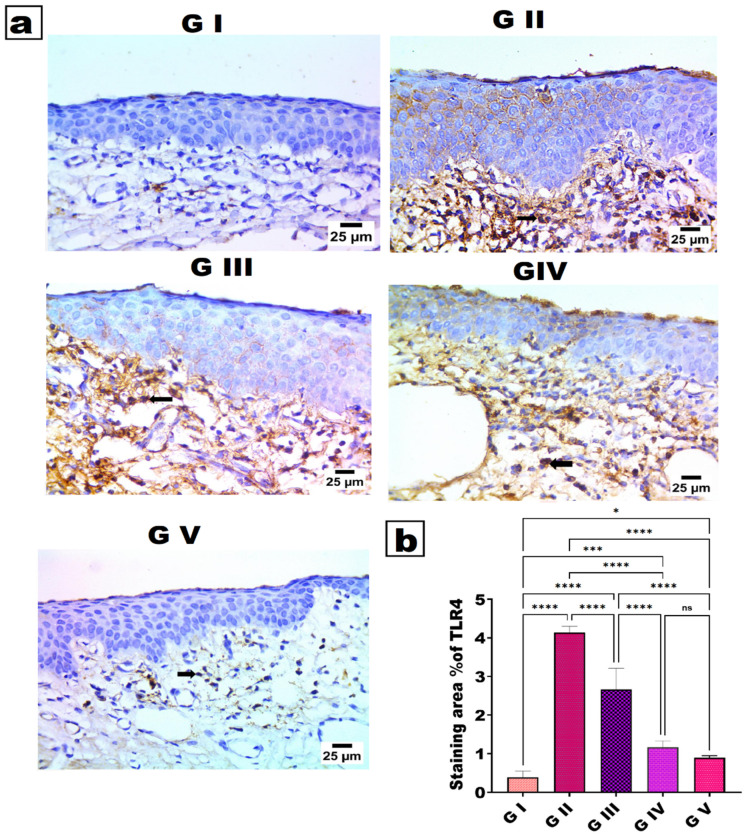
(**a**) Photomicrograph of immunohistochemical staining of TLR4 in the substantia propria of conjunctival tissues across experimental groups using the peroxidase–DAB method. Brown coloration indicates positive TLR4 expression. (**b**) Quantitative analysis of TLR4 immunostaining expressed as percentage area. Data is presented as mean ± SD. A significant increase in TLR4 expression was observed in the Diseased Group (GII) compared with the Normal Group (GI), whereas the treatment groups (GIII–GV) showed a progressive reduction, with GV restoring expression to near-normal levels (ns vs. GI). Statistical significance is indicated as * *p* < 0.05, *** *p* < 0.001, and **** *p* < 0.0001.

TLR4 immunostaining in the conjunctival substantia propria clearly links allergic inflammation to innate immune activation and shows that tacrolimus nanocarrier treatments effectively downregulate this pathway. Figure 9a shows a photomicrograph of immunohistochemical staining of TLR4; in the normal group (GI), TLR4 staining is almost absent, indicating only basal expression of this receptor in conjunctival stromal cells under physiological conditions. This is consistent with human data showing low but detectable TLR4 expression in normal conjunctival epithelium and stroma. In the diseased allergic group (GII), there is a marked increase in brown DAB-positive cells throughout the substantia propria, and the quantitative staining area rises to the highest value among all groups. This reflects strong TLR4 up-regulation in infiltrating inflammatory cells (CD4^+^ T cells, eosinophils, mast cells, and macrophages) and resident stromal cells, as reported in ocular allergic and other inflammatory ocular surface diseases where TLR4 contributes to NF-κB activation and production of IL-6, TNF-α, and other cytokines. In GIII and GIV, treatment reduces TLR4 expression compared with GII, but staining remains moderate–strong, indicating only partial suppression of the TLR4-driven inflammatory milieu. This pattern fits with reports that modulation of TLR4 signaling can attenuate, but not completely normalize, Th2-associated allergic conjunctivitis. In contrast, GV shows only mild TLR4-positive staining, with quantitative values not significantly different from GI (ns vs. GI). Histologically, only scattered brown cells remain in the substantia propria. This suggests that the optimized tacrolimus formulation (e.g., TCR-Cubs/HPMC-PVP MNs) has nearly restored the conjunctival innate immune status to normal by strongly down-regulating TLR4-mediated signaling. Given that TLR4 activation on ocular surface cells and CD4^+^ T cells promotes production of IL-1β, IL-6, and TNF-α in allergic conjunctivitis, the normalization of TLR4 in GV supports a mechanistic explanation for the observed clinical and cytokine improvements with this treatment [94,128]. Allergic conjunctivitis is associated with significant TLR4 up-regulation in conjunctival stroma, in agreement with clinical and experimental work implicating TLR4 in ocular surface inflammation; tacrolimus, especially when delivered via an optimized cubosome–microneedle system, effectively suppresses TLR4 expression back toward baseline, indicating strong control of TLR4/NF-κB–dependent innate and adaptive inflammatory responses in the conjunctiva.

## 4. Conclusions

This study concludes that cubosome-loaded dissolving microneedles are a promising ocular delivery system for the treatment of allergic conjunctivitis. The modified formulation effectively achieved sustained drug release, increased corneal penetration, and boosted overall therapeutic efficacy relative to traditional delivery methods. Treatment with the optimized cubosome–microneedle system markedly decreased key pro-inflammatory mediators, such as TNF-α, IL-1β, IL-6, and NLRP3, signifying successful attenuation of the inflammatory response. This effect was additionally corroborated at the molecular level by the downregulation of critical regulatory genes, specifically CPA3, BCL2, and TGF-β1, alongside diminished TLR4 expression and the restoration of nearly normal ocular tissue architecture in the absence of irritation. The findings underscore the dual benefits of the new system: enhanced treatment efficacy and excellent tolerability. The findings indicate that the optimized TCR-Cubs-MNs system is a viable, patient-friendly approach for managing allergic conjunctivitis and has the potential to expand future ocular drug-delivery applications.

## Figures and Tables

**Table 1:** Box–Behnken design utilized for optimizing tacrolimus-loaded cubosome formulations.

Factors (Independent Variables)	Design Levels		
	Low (−1)	Medium (0)	High (+1)
A: GMO W/W%	1.25	5	10
B: GMO: F127	5	15	25
C: Homogenization time	0	5	10
Responses (dependent variables)	Goal		
Y1: EE (%)	Maximize		
Y2: PS (nm)	Minimize		
Y3: PDI	Minimize		
Y4: ZP (mV)	Maximize		

**Abbreviations**: GMO, glyceryl monooleate; F127, Pluronic^®^ F127; EE, entrapment efficiency; PS, Particle size; PDI, polydispersity index; ZP, zeta potential.

**Table 2:** Composition of tacrolimus-loaded cubosomes with their measured responses (*n* = 3 ± SD).

Run	Factors			Responses			
	A: GMO W/W (%)	B: GMO: F127	C: Homogenization Time	PS (nm)	PDI	ZP (mV)	EE (%)
1	10	15	10	181 ± 0.65	0.34 ± 0.01	−24.8 ± 0.45	92.6 ± 0.95
2	1.25	15	10	204 ± 0.88	0.48 ± 0.04	−27 ± 0.66	82.7 ± 0.58
3	1.25	25	5	215 ± 0.54	0.52 ± 0.02	−35.5 ± 0.23	82 ± 0.63
4	10	15	0	169 ± 0.52	0.28 ± 0.09	−27.7 ± 0.46	86 ± 0.87
5	5	5	0	177 ± 0.59	0.33 ± 0.07	−27.6 ± 0.71	90.8 ± 0.61
6	10	5	5	165 ± 0.63	0.27 ± 0.05	−24.7 ± 0.45	95 ± 0.83
7	1.25	15	5	199 ± 0.68	0.45 ± 0.03	−27.7 ± 0.62	86.5 ± 0.57
8	10	25	5	182 ± 0.98	0.36 ± 0.05	−28.9 ± 0.28	90.4 ± 0.44
9	1.25	15	0	192 ± 0.59	0.42 ± 0.06	−33.4 ± 0.76	86.5 ± 0.83
10	10	5	10	176 ± 0.99	0.35 ± 0.08	−19 ± 0.92	95 ± 0.45
11	5	15	5	190 ± 0.58	0.39 ± 0.09	−31.1 ± 0.56	89.1 ± 0.87
12	5	15	5	191 ± 0.38	0.38 ± 0.02	−29.8 ± 0.83	88.6 ± 0.56
13	5	25	10	211 ± 0.67	0.51 ± 0.04	−35 ± 0.91	85 ± 0.87
14	1.25	5	5	185 ± 0.34	0.41 ± 0.01	−26.2 ± 0.56	88.3 ± 0.86
15	5	25	0	192 ± 0.65	0.47 ± 0.05	−36 ± 0.34	86.8 ± 0.23
OF	10	5	7	210 ± 0.91	0.29 ± 0.03	−21 ± 0.87	93.3 ± 0.45

**Abbreviations**: GMO, glyceryl monooleate; F127, Pluronic^®^ F127; PS, Particle size; PDI, polydispersity index; ZP, zeta potential; EE, entrapment efficiency; and OF, optimized formula.

**Table 3:** Fabrication of HPMC/PVP K90 dissolving microneedles loading freeze-dried tacrolimus-loaded cubosomes (TCR-Cubs).

Microneedles Formulations	HPMC E50 (% *w*/*w* of Total Solids)	PVP K90 (% *w*/*w* of Total Solids)
HPMC/PVP-MNs1	20	80
HPMC/PVP-MNs2	33	67
HPMC/PVP-MNs3	50	50

**Abbreviation**: HPMC, hydroxypropyl methylcellulose; PVP K90, polyvinylpyrrolidone K90; MNs, microneedles; TCR, tacrolimus; Cubs, cubosomes; TCR-Cubs, tacrolimus-loaded cubosomes.

**Table 4:** The primer sequences used for amplification of the mouse BCL2, CPA3, and TGF-β1 genes.

Primer	Sequence	NCBI Reference Sequence	Amplification Size	Annealing Temperature
*BCL2*	F: 5′-AGTTCCGTTGCCTTTTCTCC-3′ R: 5′-CAGGCTGGAGAAGTTGTTGA-3	XM_002718899.4	150	60 °C
*CPA3*	F: 5′-TGCCTGGACTACAACTTCCT-3′ R: 5′-GGAGGTAGTCGTCTCCAAGT-3′	XM_017350820.1	140	60 °C
*TGF-β1*	F: 5′-ACTGGAGTTGTACGGCAGTG-3′ R: 5′-GGGGCTGATCCCGTTGATTT-3′	XM_002719325.5	123	60 °C

**Note**: BCL2: B-cell lymphoma 2; CPA3: carboxypeptidase A3; TGF-β1: transforming growth factor, beta 1; F: forward; R: reverse.

**Table 5:** Output data of the Box–Behnken design analysis of tacrolimus-loaded cubosomes.

Responses	Y1: PS	Y2: PDI	Y3: ZP	Y4: EE%
Minimum	165	0.27	−36	82
Maximum	215	0.52	−19	95
Model	Quadratic	Linear	2F1	2F1
*F*-value	88.82	98.35	21.23	13.16
*P*-value	<0.0001	<0.0001	<0.0001	0.0009
R^2^	0.985	0.994	0.853	0.907
Adjusted R^2^	0.974	0.984	0.813	0.839
Predicted R^2^	0.928	0.949	0.719	0.583
Adequate precision	28.855	31.203	14.659	12.261
Significant factors	A, B, C	A, B, C	B	A, B

**Table 6:** Stability study for optimized tacrolimus-loaded cubosome (TCR-Cubs) formulations. Data are displayed as mean ± SD from three independent tests (*n* = 3).

Storage Time	Refrigerated Temperature (4 ± 1 °C)				Ambient Temperature			
	PS (nm)	PDI (nm)	ZP (mV)	EE (%)	PS (nm)	PDI (nm)	ZP (mV)	EE (%)
After 24 h	210 ± 0.9	0.29 ± 0.03	−21 ± 0.87	93 ± 0.45	213 ± 0.01	0.31 ± 0.03	−21 ± 0.34	92 ± 0.49
3 months	201 ± 0.5	0.34 ± 0.01	−32 ± 0.65	91 ± 0.34	204 ± 0.03	0.41 ± 0.05	−36 ± 0.27	89 ± 0.45
6 months	189 ± 0.23	0.45 ± 0.03	−35 ± 0.04	88 ± 0.61	185 ± 0.04	049 ± 0.02	−41 ± 0.34	87 ± 0.78

**Table 7:** Kinetics analysis of the release behavior of tacrolimus from the optimum tacrolimus-loaded cubosome (TCR-Cubs), its corresponding HPMC-PVP K90 microneedles loaded with optimum tacrolimus-loaded cubosome, and tacrolimus suspension.

Formulation	Zero Order (R^2^)	First Order (R^2^)	Higuchi (R^2^)	Korsmeyer– Peppas (R^2^)	n (Peppas)	Best Fit Model
Free TCR	0.72	0.78	0.93	0.96	≈0.32	Korsmeyer–Peppas (Fickian diffusion)
TCR-Cubs	0.71	0.85	0.92	0.99	≈0.50	Korsmeyer–Peppas (anomalous/non-Fickian transport)
TCR-Cubs/HPMC-PVP K90-MNs	0.65	0.94	0.89	0.97	≈0.39	First-order (diffusion-controlled from MN matrix)

**Note**: Free TCR: tacrolimus suspension; TCR-Cubs: optimum tacrolimus-loaded cubosome; Cubs/HPMC-PVP K90-MNs: HPMC-PVP K90 microneedles loaded with optimum tacrolimus-loaded cubosome.

## Data Availability

The original contributions presented in this study are included in the article. Further inquiries can be directed to the corresponding authors.

## Abbreviations

AC	Allergic Conjunctivitis
AH	Aluminum Hydroxide
ANOVA	Analysis of Variance
API	Active Pharmaceutical Ingredient
BBD	Box–Behnken Design
BCL2	BCL2-like 1/BCL2L1
CAP	Cellulose Acetate Phthalate
CAP-NFs	Cellulose Acetate Phthalate Nanofibers
CPA3	Carboxypeptidase A3
Cubs	Cubosomes
DSC	Differential Scanning Calorimetry
EE%	Entrapment Efficiency Percentage
ELISA	Enzyme-Linked Immunosorbent Assay
ER	Enhancement Ratio
FTIR	Fourier Transform Infrared Spectroscopy
GAPDH	Glyceraldehyde-3-Phosphate Dehydrogenase
GMO	Glyceryl Monooleate
H&E	Hematoxylin and Eosin
HPLC	High-Performance Liquid Chromatography
HPMC	Hydroxypropyl Methylcellulose
IHC	Immunohistochemistry
IL-1β	Interleukin-1 Beta
IL-6	Interleukin-6
LOD	Loss on Drying
MEMS	Micro-Electro-Mechanical Systems
MN	Microneedle
MNs	Microneedles
OF	Optimized Formula
OVA	Ovalbumin
PBS	Phosphate-Buffered Saline
PDI	Polydispersity Index
PS	Particle Size
PVP	Polyvinylpyrrolidone
PVA	Polyvinyl Alcohol
qRT-PCR	Quantitative Real-Time Polymerase Chain Reaction
SEM	Scanning Electron Microscopy
SLNs	Solid Lipid Nanoparticles
TCR	Tacrolimus
TEM	Transmission Electron Microscopy
TGF-β1	Transforming Growth Factor Beta-1
TLR4	Toll-Like Receptor 4
TNF-α	Tumor Necrosis Factor Alpha
ZP	Zeta Potential
VKC/AKC	Vernal Keratoconjunctivitis/Atopic Keratoconjunctivitis
